# Lipid changes during endocrine therapy in early-stage breast cancer patients: A real-world study

**DOI:** 10.1186/s12944-024-02002-6

**Published:** 2024-01-08

**Authors:** Yuechong Li, Zixi Deng, Yingjiao Wang, Songjie Shen

**Affiliations:** Department of Breast Surgery, Peking Union Medical College Hospital, Peking Union Medical College, Chinese Academy of Medical Sciences, Beijing, China

**Keywords:** Lipid profile, Breast cancer, Endocrine therapy, Toremifene, Aromatase inhibitors

## Abstract

**Background:**

Endocrine drugs may affect lipid metabolism in breast cancer (BC) patients. This study explores lipid changes in early-stage BC patients taking different endocrine drugs.

**Methods:**

The changing trend of blood lipid during endocrine therapy in 2756 BC patients from January 2013 to December 2021 was retrospectively analyzed. The changes in four lipid parameters were assessed by the Generalized Linear Mixed Model, including total cholesterol (TC), triglycerides (TG), low-density lipoprotein (LDL-C), and high-density lipoprotein (HDL-C). These parameters were quantified at baseline and at 6, 12, 18, 24, 36, 48, 60, and 72 months after endocrine therapy initiation. Furthermore, a subgroup analysis according to menopausal status or medication types was conducted.

**Results:**

A total of 1201 patients taking aromatase inhibitors (AIs), including anastrozole (ANA), letrozole (LET), or exemestane (EXE), and 1555 patients taking toremifene (TOR) were enrolled. TC and TG levels showed a significantly elevated trend during 5 years of treatment (*P* < 0.05). HDL-C levels increased from baseline in the TOR group (*P* < 0.05). Compared with the postmenopausal AI group, the increasing trends of TC, TG, and LDL-C in the premenopausal AI group were more evident with the extension of time (β = 0.105, 0.027, 0.086, respectively). Within 3 years, TC, TG, and LDL-C levels in the ANA and LET groups were significantly higher than baseline (*P* < 0.05). Moreover, the levels of TG in the EXE group were significantly lower than that in the ANA or LET group (*P* < 0.05), but this significant difference disappeared after 3 years.

**Conclusions:**

AIs significantly influenced lipid profiles more than TOR. AIs had a greater effect on blood lipids in premenopausal patients. Steroidal AIs (EXE) may affect lipid levels less than nonsteroidal AIs (ANA and LET).

**Supplementary Information:**

The online version contains supplementary material available at 10.1186/s12944-024-02002-6.

## Background

The latest global cancer burden data revealed that breast cancer (BC) has eclipsed lung cancer as the most predominant malignancy worldwide, accounting for nearly 2.26 million newly diagnosed cases globally [1]. Notably, the incidence of BC is also increasing annually in China [2]. BC with hormone receptors (HR) positive is the most common subtype, constituting approximately 60% of all cases [3], deserving urgent exploration.

Adjuvant endocrine therapy is an essential component of comprehensive treatment for patients with HR-positive BC and lasts 5–10 years. Endocrine drugs include two main types, namely selective estrogen receptor modulators (SERMs) and aromatase inhibitors (AIs). Notably, toremifene (TOR) is one type of SERM. AIs are mainly divided into nonsteroidal AIs, such as anastrozole (ANA) and letrozole (LET), and steroidal AIs as exemestane (EXE). They can inhibit the growth of BC through competitive binding with estrogen receptors or reduce estrogen levels by suppressing aromatase activity. In doing so, these therapies substantially improve the prognosis of HR-positive BC with lower recurrence rates and better overall survival [4]. However, estrogen plays a variety of physiological functions, including lipid and bone metabolism and cardiovascular, cognitive, and sexual functions [5]. Studies have also demonstrated that prolonged diminution of estrogen levels over an extended period may precipitate dyslipidemia, thereby elevating the susceptibility to cardiovascular diseases (CVD), such as myocardial infarction and stroke [6–8]. They may be even more noticeable in premenopausal BC survivors due to the abrupt suppression of estrogen [9]. In particular, CVD is estimated to be the leading cause of noncancer deaths in BC patients, especially for elderly people with early-stage BC [10]. Therefore, a comprehensive investigation of the enduring side effects of endocrine therapy is imperative.

Little consensus exists regarding the specific role of endocrine drugs in lipid metabolism. Some studies claimed that toremifene (TOR) is associated with a favorable influence on lipid profiles, with reduced triglyceride (TG) and increased high-density lipoprotein cholesterol (HDL-C) [11]. Other studies reported adverse lipid profile effects of endocrine therapy in premenopausal BC patients [12]. Additionally, a small-scale clinical trial conducted among postmenopausal Chinese BC patients indicated that nonsteroidal AIs increased the risk of lipid events [13]. Moreover, there are few studies on the impact of various endocrine drugs on lipid profiles, especially the comparisons between AIs in real-world studies. Comprehending alterations in blood lipid profiles during endocrine therapy and the impact of diverse endocrine drugs on blood lipid contributes to informed decision-making regarding endocrine drug selection for individual patients in clinical practice. Therefore, this large-scale, real-world retrospective study aims to investigate the blood lipid changes throughout endocrine therapy and assess the influence of diverse endocrine drugs on lipid metabolism.

## Methods

### Study population

**Fig. 1:**
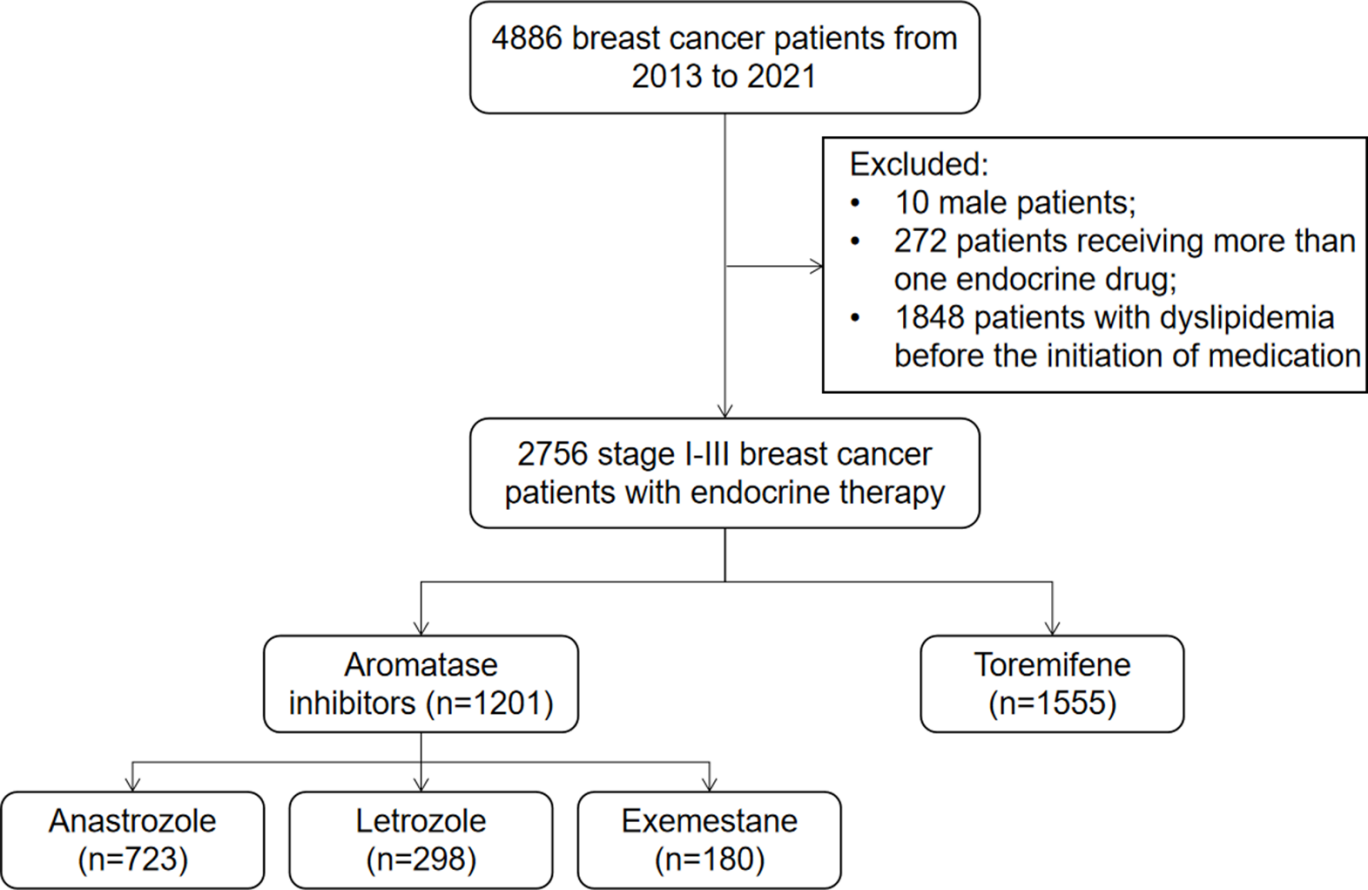
Patient selection flowchart

Using the big data query and analysis system, 4886 BC patients undergoing endocrine therapy were retrospectively enrolled between January 1, 2013, and December 31, 2021, at Peking Union Medical College Hospital (PUMCH). Ten male patients were excluded. Two hundred seventy-two patients who received multiple SERMs or AIs during endocrine therapy were excluded to avoid interference with the analysis due to alterations in endocrine drugs. Additionally, 1848 patients with dyslipidemia before the initiation of medication were excluded. Overall, 2756 stage I-III BC patients with endocrine therapy were enrolled. Figure 1 shows the patient selection flowchart. This study was approved by the Ethics Committee of PUMCH (approval number: I-22PJ227). All patients signed informed consent for related treatment. The inclusion criteria comprised the following: (1) ≥ 18-year-old female patients; (2) stage I-III BC patients; (3) patients with HR-positive BC who have completed endocrine therapy for at least 6 months; (4) patients who received letrozole (LET), anastrozole (ANA), exemestane (EXE), or TOR as endocrine therapy. The exclusion criteria comprised the following: (1) patients who changed endocrine drugs, namely patients who have taken more than one endocrine drug; (2) patients with dyslipidemia or patients who took lipid-lowering drugs prior to endocrine therapy; (3) patients who received endocrine therapy during neoadjuvant therapy or before BC diagnosis. All enrolled patients received surgical procedures and systemic therapies per the National Comprehensive Cancer Network (NCCN) guidelines. All premenopausal women who received AIs were injected with ovarian function suppression (OFS) drugs. Dyslipidemia was defined as meeting any of the following criteria: total cholesterol (TC) ≥ 5.2 mmol/L, TG ≥ 1.7 mmol/L, low-density lipoprotein cholesterol (LDL-C) ≥ 3.4 mmol/L, and HDL-C < 1.0 mmol/L [14].

### Data collection

Data collected included age at diagnosis, height, weight, body mass index (BMI), menopausal status, treatment (breast-conserving surgery or mastectomy, chemotherapy, endocrine therapy, and targeted therapy), comorbidities (hypertension, coronary heart diseases, diabetes, and fatty liver), and lipid levels at baseline and at 6, 12, 18, 24, 36, 48, 60 and 72 months after initiation of endocrine therapy. BMI was calculated as the weight divided by the square of height (Kg/m^2^). Furthermore, lipid levels include TC, TG, LDL-C, and HDL-C.

### Statistical analysis

As applicable, statistical comparisons of baseline lipid profiles and covariates among various subgroups were conducted using Pearson’s chi-square, Fisher’s exact, Student’s t-tests, or Analysis of Variance (ANOVA) tests.

The Generalized Linear Mixed Model was employed to assess alterations in lipid profiles across various endocrine therapy groups at different administration time points. Model-adjusted least-square means were also used to describe the blood lipid levels in different subgroups. All tests were two-sided. *P* < 0.05 was considered statistically significant. Statistical analyses were performed using SPSS 25.0 statistical software (IBM Corporation, Armonk, NY, USA).

## Results

### Comparison of BC patients taking AIs or TOR

A total of 2756 patients were included: 1201 patients taking AIs and 1555 patients taking TOR. The baseline characteristics are shown in Table 1. The average age and BMI in the AI group were higher than in the TOR group (*P* < 0.001). Furthermore, the proportion of patients in the TOR group undergoing breast-preserving surgery was higher than that of the AI group (*P* < 0.001). In the AI group, the baseline TG levels were higher than the TOR group (*P* < 0.001).

**Table 1:** Baseline characteristics of BC patients taking AIs or TOR

Variables	AIs (*n* = 1201)	TOR (*n* = 1555)	P^#^
Age, mean(SD)	55.61 (11.89)	42.2 (6.71)	< 0.001
BMI(SD)	24.18 (3.65)	22.93 (3.42)	< 0.001
**Surgery**			< 0.001
Breast conserving	486 (40.5)	762 (49.0)	
Mastectomy	680 (56.6)	736 (47.3)	
**Adjuvant therapy**			
Target therapy	202 (16.8)	139 (8.9)	< 0.001
Chemotherapy	542 (45.1)	430 (27.7)	< 0.001
**Comorbidities**			
Hypertension	179 (14.9)	233 (15.0)	0.954
Coronary diseases	14 (1.2)	24 (1.5)	0.399
Diabetes	51 (4.2)	83 (5.3)	0.187
Fatty liver	1 (0.1)	5 (0.3)	0.241
**Baseline lipid profiles**			
TC, mean (SD)	4.41 (0.53)	4.37 (0.50)	0.132
TG, mean (SD)	0.95 (0.34)	0.81 (0.32)	< 0.001
LDL-C, mean (SD)	2.46 (0.51)	2.44 (0.46)	0.352
HDL-C, mean (SD)	1.42 (0.25)	1.43 (0.25)	0.122

^#^Comparisons of surgery type, adjuvant therapy, and comorbidities are conducted using Pearson’s chi-square or Fisher’s exact; comparisons of age, BMI, and baseline lipid profiles are conducted using Student’s t-tests

**Fig. 2:**
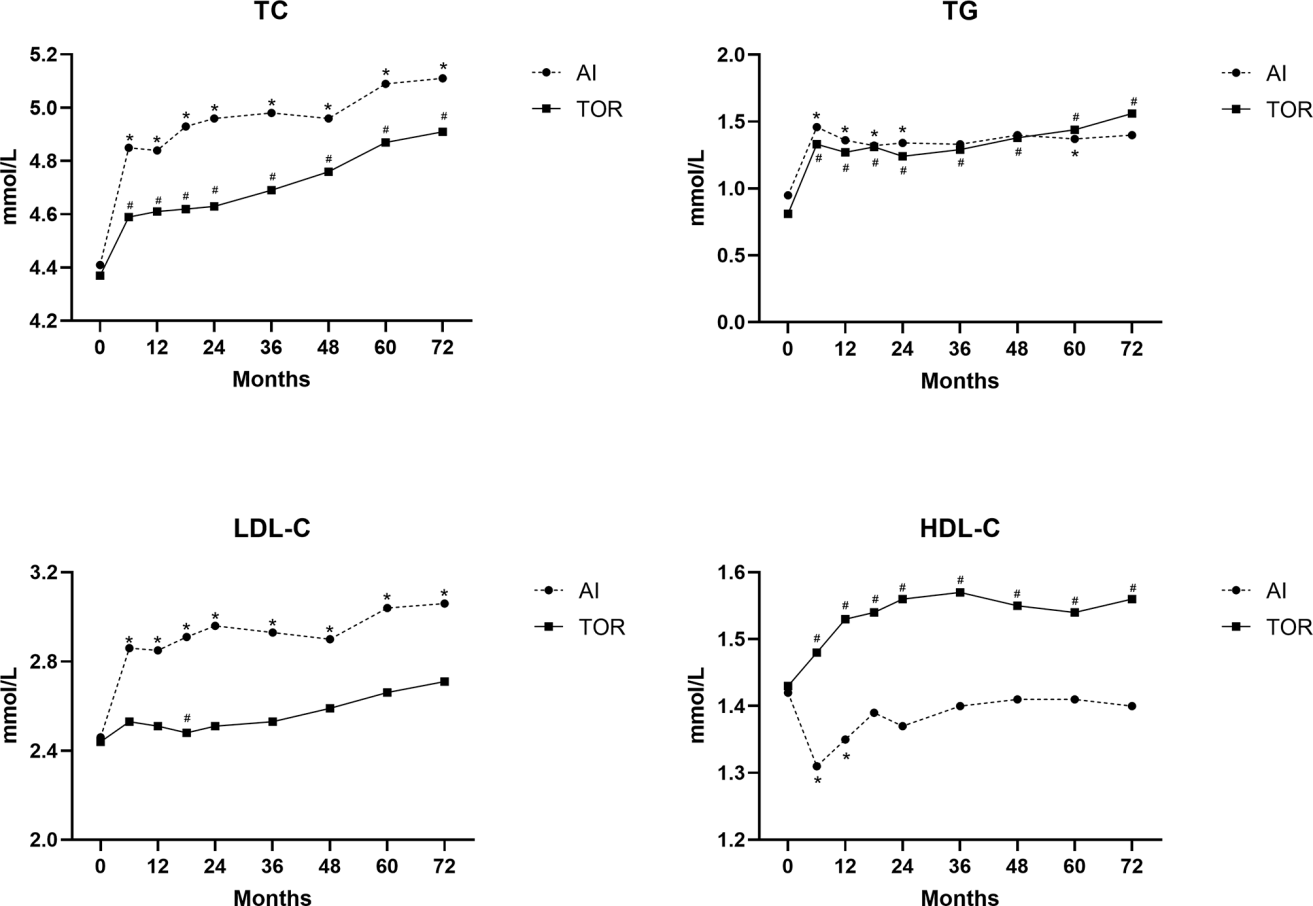
Changes of lipid profiles in AI and TOR groups compared with baseline using Student’s t-tests; * represents *P* < 0.05 in the AI group; #represents *P* < 0.05 in the TOR group

The changing trend of different lipid indexes in each group over time is shown in Fig. 2. Compared with the baseline of each group, TC and TG levels were significantly higher at 6 months after taking the drug (*P* < 0.05), and an upward trend existed during the subsequent 5 years of treatment (*P* < 0.05). Compared with baseline, LDL-C levels in the AI group significantly increased (*P* < 0.05), while the TOR group showed an increasing trend with no significant difference. HDL-C levels in the AI group significantly decreased from baseline levels at 6 months and 1 year after medication (*P* < 0.05), and those in the TOR group increased from baseline levels (*P* < 0.05).

In general, TC, TG, and LDL-C levels showed an increasing trend with the extension of medication time (β = 0.063, 0.054, 0.057, respectively) (Table 2). Compared with the AI group, TC and LDL-C levels in the TOR group increased less significantly with the extension of time (β=-0.020, -0.055, respectively). Furthermore, TC and TG levels exhibited positive associations with age (β = 0.005, 0.006, respectively), and TG and LDL-C levels exhibited positive associations with BMI (β = 0.031, 0.017, respectively). In contrast, HDL-C levels exhibited negative associations with BMI (β=-0.027).

**Table 2:** Comparison of lipid profiles in BC patients taking AIs or TOR

	TC (β^#^, 95%CI)	TG (β^#^, 95%CI)	LDL-C (β^#^, 95%CI)	HDL-C (β^#^, 95%CI)
**Endocrine therapy**				
AIs	0	0	0	0
TOR	-0.015 (-0.118,0.089)	-0.034 (-0.101, 0.033)	0.008 (-0.083,0.099)	0.034 (-0.006,0.074)
Time	0.063 (0.052,0.074)	0.054 (0.043,0.064)	0.057 (0.047,0.067)	0.009 (0.005,0.012)
Time*endocrine therapy				
AIs*time	0	0	0	0
TOR*time	-0.020 (-0.035, -0.005)	0.033 (0.018,0.047)	-0.055 (-0.069, -0.042)	0.020 (0.014,0.025)
Age	0.005 (0.001,0.009)	0.006 (0.004,0.009)	0.001 (-0.002,0.005)	0.001 (-0.001,0.002)
BMI	-0.005 (-0.015,0.006)	0.031 (0.024,0.039)	0.017 (0.008,0.026)	-0.027 (-0.031, -0.023)
**Surgery**				
Breast conserving	0	0	0	0
Mastectomy	0.167 (0.096,0.237)	0.068 (0.019,0.117)	0.151 (0.088,0.213)	-0.030 (-0.058, -0.003)
**Adjuvant therapy**				
Target therapy	0.079 (-0.040,0.198)	-0.001 (-0.082,0.081)	0.076 (-0.029, 0.182)	-0.001 (-0.048,0.045)
Chemotherapy	-0.007 (-0.093,0.079)	-0.055 (-0.114,0.004)	0.006 (-0.070,0.082)	0.024 (-0.010,0.058)
**Comorbidities**				
Hypertension	-0.012 (-0.110,0.087)	-0.007 (-0.075,0.061)	0.019 (-0.068,0.106)	-0.006 (-0.045,0.033)

^#^Generalized Linear Mixed Models are used; the administration time was taken as a continuous variable

Subsequently, the lipid profiles were compared at each assessment point in time (Supplementary Table 1). In the AI group, LDL-C levels were significantly higher than in the TOR group during 5 years of endocrine therapy (*P* < 0.05). TC and LDL-C levels in the AI group were significantly higher than those in the TOR group from 18 months to 3 years after medication (*P* < 0.05). Furthermore, the levels of HDL-C in the TOR group were significantly higher than those in the AI group during the medication time (*P* < 0.05).

### Comparison of premenopausal and postmenopausal patients taking AIs

Next, subgroup analysis was conducted for patients taking AIs according to menopause status. The baseline characteristics are shown in Supplementary Table 2. Of the 1201 patients taking AIs, 889 were postmenopausal, and 312 were premenopausal. Notably, postmenopausal patients’ mean age and BMI were higher than premenopausal patients (*P* < 0.001).

**Fig. 3:**
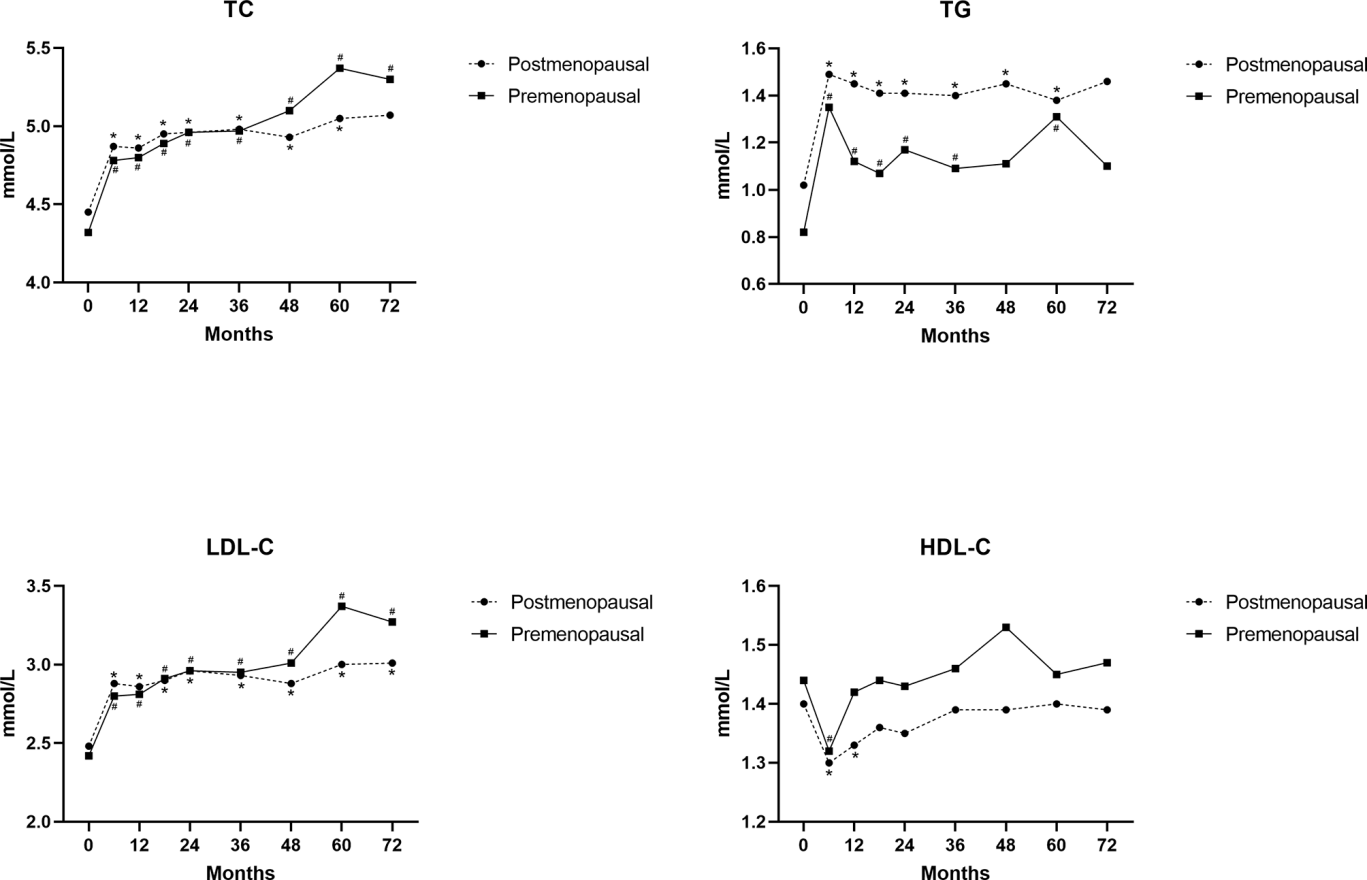
Changes of lipid profiles in premenopausal and postmenopausal groups compared with baseline using Student’s t-tests; * represents *P* < 0.05 in the postmenopausal group; # represents *P* < 0.05 in the premenopausal group

Compared with the baseline lipid profiles, the levels of TC and LDL-C in each group showed a significant increasing trend (*P* < 0.05) (Fig. 3). The TG levels in the two groups peaked at 6 months after receiving therapy and were significantly higher than baseline (*P* < 0.05). At 12, 18, 24, 36, and 60 months after medication, TG levels were still higher in the two groups than baseline (*P* < 0.05). Moreover, the HDL-C levels in both groups decreased significantly at 6 months after medication (*P* < 0.05). Following 12 months of medication, the HDL-C levels in both groups were not significantly different from baseline levels (*P*＞0.05).

Compared with the postmenopausal AI group, the increasing trends of TC, TG, and LDL-C in the premenopausal AI group were more evident with the extension of time (β = 0.105, 0.027, 0.086, respectively) (Table 3). Similarly, TG levels exhibited positive associations with BMI (β = 0.030), and TC and HDL-C levels exhibited negative associations with BMI (β=-0.019, -0.023, respectively). Compared with the postmenopausal AI group, the levels of TC and LDL-C in the premenopausal AI group were lower within 1.5 years after medication (*P* < 0.05) (Supplementary Table 3).

**Table 3:** Comparison of blood lipids in premenopausal and postmenopausal patients taking AIs

	TC (β^#^, 95%CI)	TG (β^#^, 95%CI)	LDL-C (β^#^, 95%CI)	HDL-C (β^#^, 95%CI)
**Menopausal status**				
postmenopausal	0	0	0	0
premenopausal	-0.721 (-0.921, -0.522)	-0.264 (-0.394, -0.134)	-0.611 (-0.787, -0.435)	0.012 (-0.063,0.087)
Time	0.037 (0.024,0.051)	0.032 (0.022,0.041)	0.034 (0.023,0.046)	0.009 (0.005,0.014)
Time*menopausal status				
Postmenopausal*time	0	0	0	0
Premenopausal*time	0.105 (0.079,0.130)	0.027 (0.009,0.045)	0.086 (0.064, 0.108)	0.002 (-0.006,0.010)
Age	-0.009 (-0.016, -0.003)	0.001 (-0.004,0.005)	-0.011 (-0.017, -0.005)	-0.001 (-0.003,0.002)
BMI	-0.019 (-0.034, -0.005)	0.030 (0.020,0.040)	-0.001 (-0.013,0.012)	-0.023 (-0.028, -0.017)
**Surgery**				
Breast conserving	0	0	0	0
Mastectomy	0.181 (0.075,0.287)	0.095 (0.023,0.167)	0.162 (0.068,0.256)	-0.037 (-0.077,0.002)
**Adjuvant therapy**				
Target therapy	0.034 (-0.125,0.192)	0.007 (-0.099,0.114)	-0.006 (-0.146,0.134)	0.010 (-0.049,0.069)
Chemotherapy	0.071 (-0.059,0.201)	-0.026 (-0.114,0.063)	0.118 (0.003,0.234)	-0.014 (-0.062,0.035)
**Complication**				
Hypertension	-0.061 (-0.208,0.086)	-0.037 (-0.136,0.063)	-0.033 (-0.164,0.097)	-0.001 (-0.055,0.055)

^#^Generalized Linear Mixed Models are used; the administration time was taken as a continuous variable

### Comparison of BC patients taking different AIs

**Fig. 4:**
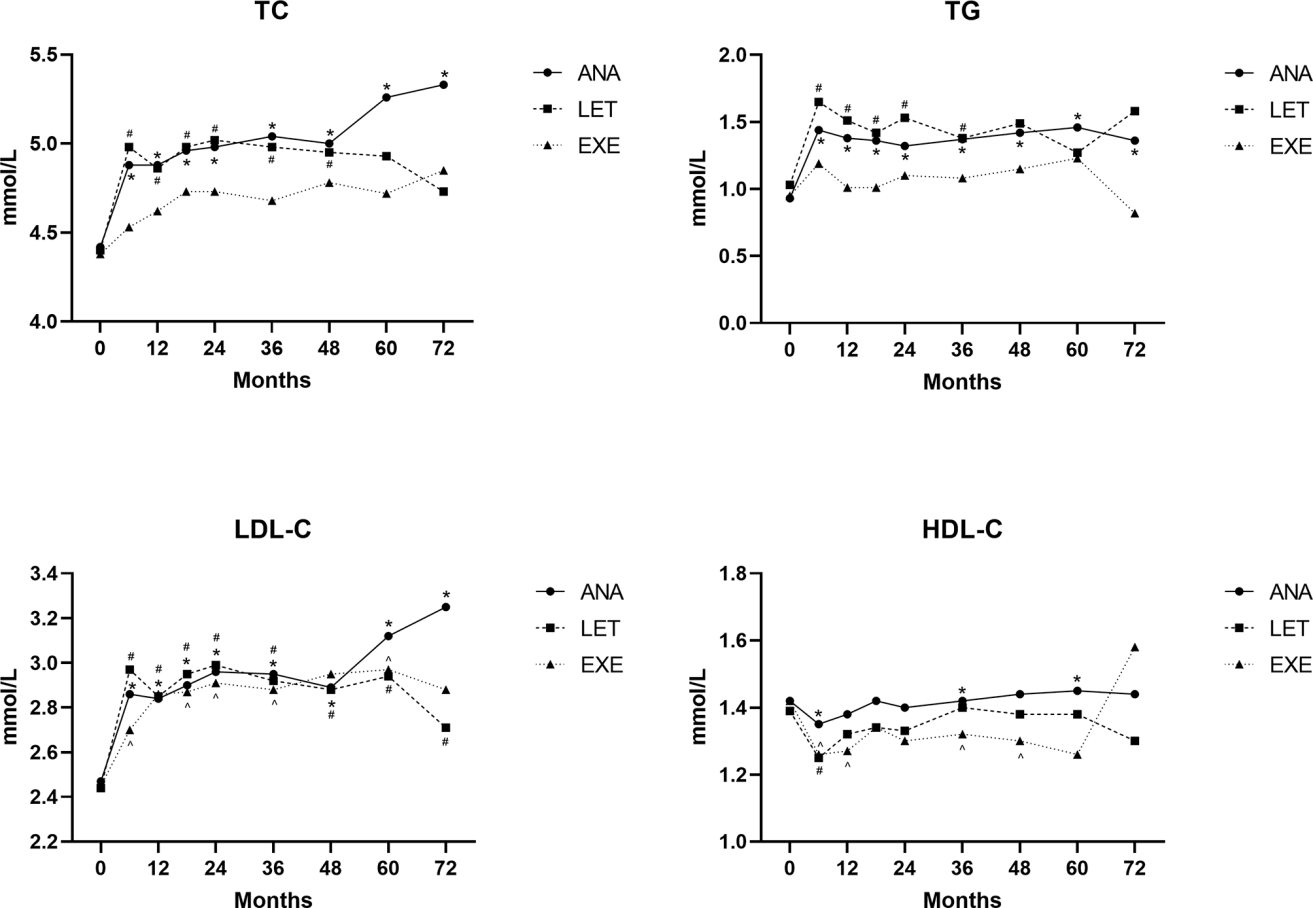
Changes of lipid profiles among different AI groups compared with baseline using Student’s t-tests; * represents *P* < 0.05 in the ANA group; # represents *P* < 0.05 in the LET group; ^ represents *P* < 0.05 in the EXE group

Among 1201 patients taking AIs drugs, 723 received ANA treatment, 298 received LET treatment, and 180 received EXE treatment. The baseline characteristics are shown in Supplementary Table 4. The changing trend of lipid profiles in each group is shown in Fig. 4. During the 3 years after taking the drugs, the TC, TG, and LDL-C levels in the ANA and LET groups were significantly higher than baseline levels (*P* < 0.05). The levels of TC and TG in the EXE group were not significantly different from the baseline. In contrast, LDL-C levels in the EXE group were significantly higher than baseline levels (*P* < 0.05). At 6 months after taking the drugs, HDL-C levels in all groups were significantly lower than baseline levels (*P* < 0.05). After that, HDL-C levels in the ANA and LET groups were not significantly different from the baseline, with the curve gradually flattened. At 12, 36, and 48 months after taking EXE, HDL-C levels were significantly lower than baseline (*P* < 0.05).

With the extension of time, the TC, TG, and LDL-C levels in different AIs groups showed an increasing trend (Supplementary Table 5). Additionally, BMI was positively correlated with TG (β = 0.030) but was negatively correlated with HDL-C and TC levels (β=-0.019, -0.023, respectively).

Overall, the main difference in the effects of different AIs on lipid levels was between EXE and nonsteroidal AIs (Table 4). The TG level in the EXE group was lower within 3 years after medication than in the steroidal AIs groups (*P* < 0.05). In contrast with the nonsteroidal AI groups, the levels of TC were lower in the EXE group at 12, 18, 36, and 48 months after medication (*P* < 0.05). Furthermore, compared with the ANA group, HDL-C levels were lower in the EXE group from 12 months to 60 months after medication (*P* < 0.05).

**Table 4:** Comparison of blood lipids in BC patients taking different AIs at each medication time

Months		ANA^&^(*n* = 723)	LET^&^(*n* = 298)	EXE^&^(*n* = 180)	ANA vs. LET P^#^	ANA vs. EXE P^#^	LET vs. EXE P^#^
**TC**							
	6	4.87 (0.08)	4.96 (0.11)	4.65 (0.14)	0.477	0.140	0.066
	12	4.86 (0.07)	4.80 (0.10)	4.40 (0.12)	0.566	< 0.001	0.007
	18	4.87 (0.07)	4.97 (0.10)	4.55 (0.12)	0.373	0.009	0.004
	24	4.98 (0.06)	4.85 (0.09)	4.61 (0.12)	0.178	0.003	0.084
	36	5.03 (0.07)	4.94 (0.10)	4.59 (0.14)	0.400	0.002	0.029
	48	4.91 (0.08)	4.82 (0.11)	4.42 (0.15)	0.468	0.003	0.023
	60	5.08 (0.11)	4.79 (0.13)	4.69 (0.22)	0.072	0.113	0.707
	72	5.03 (0.15)	4.77 (0.17)	4.55 (0.41)	0.244	0.266	0.617
**TG**							
	6	1.42 (0.06)	1.56 (0.09)	1.05 (0.10)	0.153	0.001	< 0.001
	12	1.37 (0.05)	1.40 (0.08)	0.91 (0.10)	0.752	< 0.001	< 0.001
	18	1.35 (0.06)	1.35 (0.09)	1.01 (0.10)	0.964	0.002	0.008
	24	1.26 (0.05)	1.37 (0.08)	1.00 (0.10)	0.212	0.015	0.002
	36	1.33 (0.05)	1.26 (0.08)	0.98 (0.11)	0.448	0.004	0.038
	48	1.24 (0.06)	1.19 (0.08)	1.02 (0.11)	0.614	0.085	0.212
	60	1.26 (0.07)	1.18 (0.09)	1.10 (0.15)	0.484	0.351	0.656
	72	1.42 (0.13)	0.97 (0.15)	0.89 (0.34)	0.017	0.141	0.815
**LDL-C**							
	6	2.86 (0.07)	2.94 (0.10)	2.78 (0.12)	0.470	0.554	0.287
	12	2.80 (0.06)	2.82 (0.09)	2.67 (0.10)	0.904	0.225	0.256
	18	2.81 (0.06)	2.95 (0.09)	2.77 (0.10)	0.142	0.728	0.158
	24	2.92 (0.06)	2.87 (0.08)	2.80 (0.11)	0.581	0.264	0.536
	36	2.94 (0.06)	2.91 (0.09)	2.79 (0.12)	0.810	0.243	0.374
	48	2.85 (0.07)	2.84 (0.09)	2.61 (0.13)	0.936	0.085	0.124
	60	2.96 (0.10)	2.82 (0.12)	2.86 (0.20)	0.343	0.644	0.873
	72	2.87 (0.13)	2.91 (0.15)	2.73 (0.36)	0.849	0.694	0.630
**HDL-C**							
	6	1.33 (0.02)	1.27 (0.03)	1.32 (0.04)	0.056	0.796	0.230
	12	1.39 (0.02)	1.31 (0.03)	1.26 (0.04)	0.022	0.001	0.208
	18	1.40 (0.02)	1.34 (0.03)	1.29 (0.04)	0.041	0.002	0.235
	24	1.44 (0.02)	1.35 (0.03)	1.34 (0.04)	0.004	0.007	0.702
	36	1.46 (0.02)	1.40 (0.03)	1.33 (0.04)	0.077	0.003	0.140
	48	1.47 (0.02)	1.40 (0.03)	1.31 (0.04)	0.024	< 0.001	0.051
	60	1.50 (0.03)	1.42 (0.04)	1.33 (0.06)	0.056	0.004	0.149
	72	1.46 (0.04)	1.38 (0.05)	1.38 (0.11)	0.211	0.471	0.955

^#^Generalized Linear Mixed Models were used to compare the blood lipids of different endocrine agents at each timepoint, taking the medication time as a classified variable

^&^Serum lipid levels were presented as model-adjusted least-square means

**Fig. 5:**
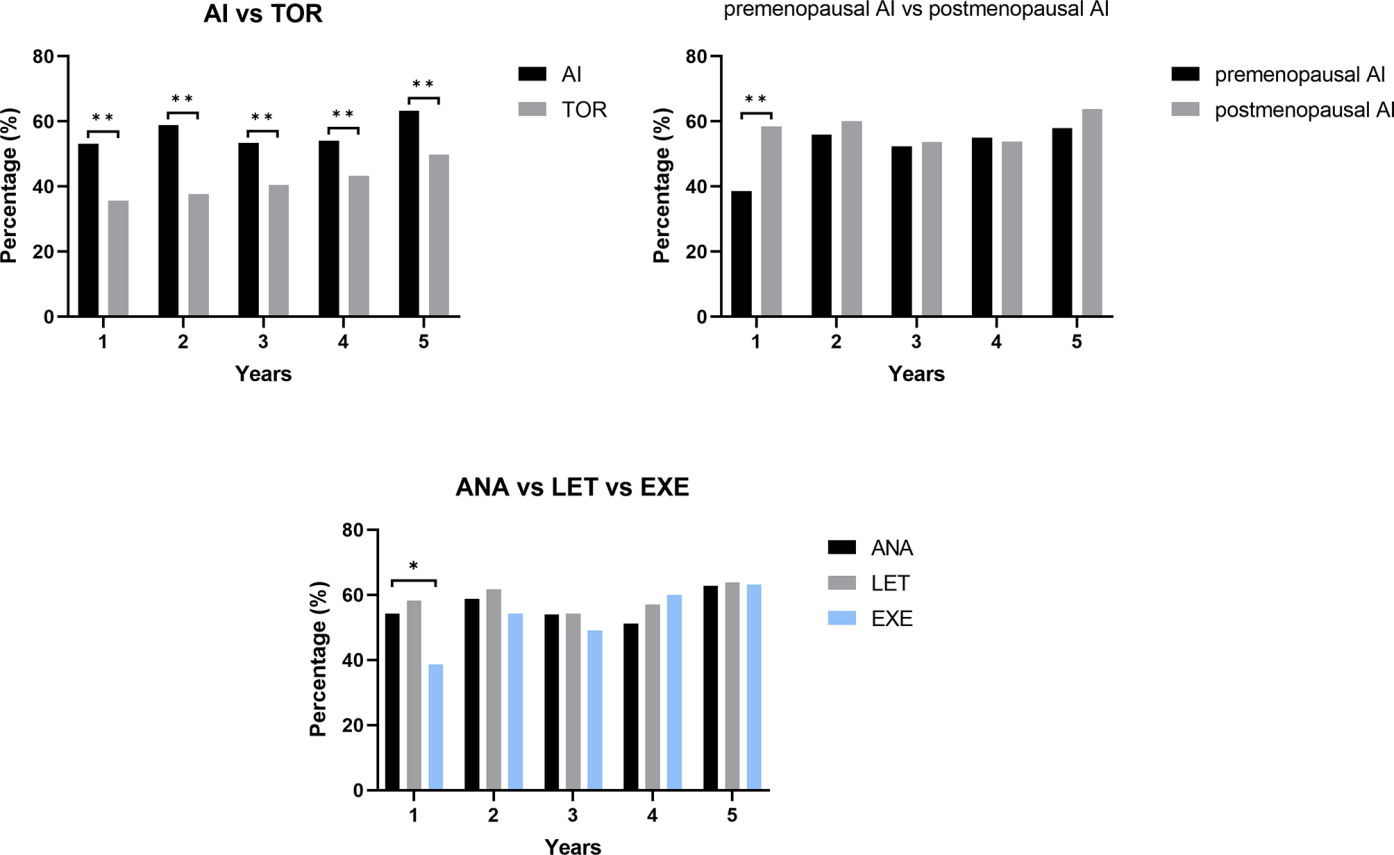
Comparison of the proportion of dyslipidemia in different groups using Pearson’s chi-square. ** represents *P* < 0.01, * represents *P* < 0.05

Finally, the proportion of dyslipidemia in each group was compared (Fig. 5). During the five years of endocrine therapy, the proportion of dyslipidemia in the AI group was significantly higher than in the TOR group (*P* < 0.01). One year after the initiation of medication, there was a higher proportion of dyslipidemia in the postmenopausal group than in the premenopausal group (*P* < 0.01). Moreover, there was a lower proportion of dyslipidemia in those taking EXE than those in nonsteroidal AI groups (*P* < 0.05).

## Discussion

TOR exhibited favorable effects on HDL-C levels in this real-world study in China, involving a long follow-up time and a large sample size. At the same time, AIs negatively influenced TC, TG, and LDL-C levels. For subgroup analysis, AIs may significantly affect lipid profiles in premenopausal and postmenopausal BC patients, with a more evident increasing trend of TC, TG, and LDL-C in the premenopausal AI group. EXE tended to have a minor effect on the levels of TC and TG compared to ANA and LET.

TOR, another type of SERM, has demonstrated comparable efficacy to tamoxifen (TAM) in HR-positive BC patients [15, 16]. It is widely used in China, so this study explored its effects on lipid profiles. The previous findings regarding the effects of TAM on blood lipids remain unclear. A randomized controlled study showed TAM lowered LDL-C and TC levels but with a small sample size and short follow-up time [17]. Other research demonstrated that TAM increased TG and LDL-C levels and was associated with more severe fatty liver disease and liver fibrosis [11, 18]. Song D et al. found that TOR improved the lipid profiles of premenopausal BC patients [11]. In this study, compared with baseline, the levels of TC and TG showed a significant increasing trend. LDL-C levels in the TOR group also showed an upward trend but no significant difference. Simultaneously, HDL-C levels increased in the TOR group. Therefore, TOR may have a protective effect on HDL-C levels. However, it may still adversely affect the levels of TC and TG.

Regarding comparing the influences of AIs and TOR on lipid profiles, AIs negatively affected TC, TG, and LDL-C levels, which tended to have a greater impact on lipid profiles than TOR. These findings are consistent with previous studies. A randomized study demonstrated that the levels of TC and LDL-C were lower following TOR treatment than after LET treatment in a prospective clinical trial [19]. A 100-month follow-up of the ATAC trial revealed a higher incidence of hypercholesterolemia in the ANA group compared to the TAM group among postmenopausal BC patients [20]. A large cohort study also found that, whether postmenopausal or premenopausal, TC and LDL-C levels in the AI group were higher than in the SERM group [21]. This difference may be due to the different mechanisms between AIs and SERMs. AIs inhibit estrogen synthesis by reducing systemic aromatization, thus weakening the favorable effects of estrogen in lipid metabolism [22]. Moreover, the structure of SERM is similar to that of estrogen, which competes with estradiol to form a stable complex with estrogen receptors. As a result, SERM can perform an estrogenic function that positively affects blood lipids to some extent [23]. Previous studies have demonstrated that high TG levels and low HDL-C levels are important risk factors for CVD [24, 25]. Therefore, for patients at high risk of CVD, TOR may be a preferred option. It is necessary to closely monitor lipid profiles during endocrine therapy, especially for those patients taking AIs. However, there is a lack of large-scale prospective studies regarding whether TOR has a protective effect on lipid metabolism, which needs further exploration.

As the increasing trends of TC, TG, and LDL-C levels in the premenopausal AI group became more apparent, premenopausal patients should pay more attention to blood lipid changes. Moreover, clinicians need to guide the management of blood lipids during patients’ follow-up visits. Notably, the abrupt suppression of estrogen in premenopausal patients may be the main reason. Research has indicated that using OFS at a younger age may exacerbate lipid-related events in premenopausal patients [26]. However, a 5-year cohort study revealed a higher incidence of dyslipidemia in postmenopausal patients compared to their premenopausal counterparts (42.6% vs. 32.6%) [21]. This study also showed a higher incidence of dyslipidemia in postmenopausal patients, especially in the first year after medication, then the difference disappeared. This may be related to the higher baseline levels of blood lipids in postmenopausal patients, making postmenopausal patients more prone to dyslipidemia. The use of lipid-lowering drugs may also affect the proportion of dyslipidemia. This finding can also likely be explained by other independent risk factors for female dyslipidemia, like BMI and age [27]. These factors may interact with each other and be associated with a poor prognosis of BC. A recent study showed that obesity was associated with an increased risk of BC recurrence among postmenopausal BC patients treated with AIs [28]. Furthermore, studies showed that dyslipidemia was associated with adverse outcomes in BC patients treated with endocrine therapy [29, 30].

Regarding comparing different AIs on lipid profiles, EXE appeared to have a smaller effect on lipid levels than nonsteroidal AIs (ANA and LET). This result is in accordance with previous studies. The MA.27 study showed that the increase of TG and TC levels was more prevalent in the ANA group than in the EXE group [31]. Wang et al. also demonstrated that compared with the steroidal AI group, the nonsteroidal AI group developed a higher cumulative incidence of dyslipidemia [13]. In this study, the levels of HDL-C in the EXE group decreased significantly. This is in accordance with a small sample size study, which showed that HDL-C levels in the EXE group decreased significantly than the placebo group at 3, 6, and 12 months [32]. A possible reason may be the metabolite of EXE, 17-hydroxy exemestane, which enhances the efficacy of suppressing aromatase [33]. Nevertheless, a prospective study in Japan showed that EXE and nonsteroidal AIs did not significantly affect lipid profiles [32, 34]. This study further showed that such differences between steroidal and nonsteroidal AIs may disappear 3 years after medication. Notably, there may be a delayed effect of lipid changes for EXE. A two-year cohort study [32] showed that the EXE group displayed no significant differences in percent change in LDL or TG at any time compared with the placebo group. The use of lipid-lowering drugs may also play a role. Thus, systematic reviews and large-scale prospective trials are warranted [35].

For subgroup analysis of patients taking AIs, significant changes in TG and HDL-C levels could be seen almost 6 months after taking the drug. However, since then, the degree of changes in these two parameters has decreased slightly. This phenomenon may be related to the prescription of lipid-lowering drugs or the clinical doctors’ advice to control lipid levels by changing lifestyle after the first follow-up of dyslipidemia. Studies have shown that lifestyle changes can help people control their lipid levels [36]. From this point of view, apart from the effects of lipid-lowering drugs, the conclusions of this study may also enlighten us to recommend a lifestyle adjustment to control blood lipid levels in the postoperative instruction, which will be more beneficial to the control of blood lipid in the entire endocrine treatment.

## Study strengths and limitations

Different endocrine drugs exhibit varying impacts on blood lipid profiles. Therefore, in the clinical treatment of women with dyslipidemia or elevated cardiovascular risk, preference should be given to endocrine drugs with minimal effects on blood lipids. This large-scale, real-world study systematically investigated changing trends in blood lipid profiles during endocrine therapy and conducted comparative analyses of the influence of diverse endocrine drugs on blood lipids. Such insights are paramount for the judicious selection of endocrine therapies tailored to individual patients in clinical practice. For instance, while AIs generally demonstrate superior overall efficacy compared to SERM, this distinction diminishes for women at low risk of BC [37]. Considering SERM may be more appropriate in cases involving women at a high risk of cardiovascular disease. Among AIs, efficacy remains consistent, but distinctions emerge in their effects on lipid profiles. Moreover, patient comorbidities should inform AI selection decisions. There exist some limitations. This study is retrospective, single-center, and lacks prognostic data regarding CVD, fatty liver, and other events. In addition, there is a lack of data on other factors influencing lipid levels, such as lipid-lowering drugs, BMI changes during the treatment, daily calorie intake, consumption, and lifestyle. The mechanism underlying the endocrine drugs affecting blood lipids is still unclear. Therefore, prospective randomized controlled trials on the influences of endocrine therapy on blood lipids and a deeper exploration of how endocrine drugs affect blood lipids are needed.

## Conclusion

In conclusion, compared with TOR, AIs tended to have a greater influence on lipid profiles. The increasing trends of TC, TG, and LDL-C levels were more evident in the premenopausal AI group, and EXE may have a minor effect on lipid levels than nonsteroidal AIs in the short term. Research into tools for assessing endocrine drugs’ impacts on lipid profiles and comorbidities should be considered.

### Electronic supplementary material

Below is the link to the electronic supplementary material.

**Supplementary Material 1:** Certificate of language-20230914

**Supplementary Material 2:** iThenticate-proof report

**Supplementary Material 3:** Supplementary tables 1-5

**Supplementary Material 4:** New certificate of language-20231225

## Data Availability

The data utilized and examined in this study are available upon reasonable request from the corresponding author.

## Abbreviations

AI: Aromatase inhibitors
ANA: Anastrozole
ANOVA: Analysis of Variance
BC: Breast cancer
BMI: Body mass index
CVD: Cardiovascular diseases
EXE: Exemestane
HDL-C: High-density lipoprotein
HR: Hormone receptor
LDL-C: Low-density lipoprotein
LET: Letrozole
NCCN: National Comprehensive Cancer Network
OFS: Ovarian function suppression
PUMCH: Peking Union Medical College Hospital
SERM: Selective estrogen receptor modulators
TC: Total cholesterol
TG: Triglycerides
TOR: Toremifene
