# Supplementary material for: Lipid changes during endocrine therapy in early-stage breast cancer patients: A real-world study
Source: Lipids Health Dis. 2024 Jan 8;23:9. doi: 10.1186/s12944-024-02002-6 (PMC10773127; doi:10.1186/s12944-024-02002-6)
Supplement: Supplementary file 1 — Supplementary Material 1: Certificate of language-20230914 [file 12944_2024_2002_MOESM1_ESM.pdf]

Language Assistant Certificate

This document is to certify that the article below has been edited by professional editors at AiMi (Scientific Editing Experts, United States. AiMi Academic Service, LLC) to ensure that the language is clear and free of errors. The intent of the author's message was not altered in anyway during the editing process. We guarantee the quality of our editing services, with the assumption that our suggested changes have been accepted and have not been further altered without the knowledge of our editors.

TITLE OF THE MANUSCRIPT

Lipid changes during endocrine therapy in early-stage breast cancer patients: a real-world study

AUTHORS

Yuechong Li, Zixi Deng, Yingjiao Wang, Songjie Shen

Kathleen Frey

Vice Chairman

Date: Sep 14, 2023

AiMi, offers professional English language editing and publication support services to authors engaged in over 400 areas of research through its community of experienced editors, which includes doctors, published scientists, and researchers with peer review experience. Authors who work with AiMi are guaranteed excellent language quality and timely delivery.

Contact AiMi:
AiMi Academic Services, LLC
3338 Bradbury Rd, Madison, WI, 53719
www.aimieditor.com
inquiry@aimieditor.com
