# Supplementary material for: Lipid changes during endocrine therapy in early-stage breast cancer patients: A real-world study
Source: Lipids Health Dis. 2024 Jan 8;23:9. doi: 10.1186/s12944-024-02002-6 (PMC10773127; doi:10.1186/s12944-024-02002-6)
Supplement: Supplementary file 2 — Supplementary Material 2: iThenticate-proof report [file 12944_2024_2002_MOESM2_ESM.pdf]

iThenticate®

Lipid changes during endocrine therapy.docx
Dec 16, 2023
6056 words / 34538 characters

Lipid changes during endocrine therapy

Sources Overview

20%
OVERALL SIMILARITY

1	www.frontiersin.org<br>INTERNET	3%
2	www.ncbi.nlm.nih.gov<br>INTERNET	2%
3	zenodo.org<br>INTERNET	1%
4	Preprint source<br>www.researchsquare.com<br>INTERNET	1%
5	SHUANG-LI XIONG. "EFFECTS OF ORAL CHONDROITIN SULFATE ON LIPID AND ANTIOXIDANT METABOLISMS IN RATS FED A HIGH-F...<br>CROSSREF	<1%
6	www.nature.com<br>INTERNET	<1%
7	Preprint source<br>Junren Wang, Jin Yin, Jiajun Qiu, Jingwen Jiang, Yao Hu, Hong Zheng, Ting Luo, Xiaorong Zhong. "Distribution of dyslipidemia in Chine...<br>CROSSREF POSTED CONTENT	<1%
8	bmccancer.biomedcentral.com<br>INTERNET	<1%
9	www.mdpi.com<br>INTERNET	<1%
10	www.science.gov<br>INTERNET	<1%
11	publichealth.jmir.org<br>INTERNET	<1%
12	Preprint source<br>Xu Guan, Shuai Jiao, Haitao Niu, Xiaolong Ma et al. "The tumor characteristics, treatment strategy and prognosis in colorectal cancer ...<br>CROSSREF POSTED CONTENT	<1%
13	appliedresearch.cancer.gov<br>INTERNET	<1%
14	assets.researchsquare.com<br>INTERNET	<1%
15	stacks.cdc.gov<br>INTERNET	<1%
16	Torng, P.L.. "Effects of menopause and obesity on lipid profiles in middle-aged Taiwanese women: the Chin-Shan Community Cardiova...<br>CROSSREF	<1%

17 Yutaka Yamamoto, Hirotaka Iwase. "Safety profiles of aromatase inhibitors and selective estrogen-receptor modulators in the treatme...
CROSSREF
<1%

18 Linda Cucciniello, Giovanna Garufi, Rossana Di Rienzo, Claudia Martinelli et al. "Estrogen deprivation effects of endocrine therapy in br...
CROSSREF
<1%

19 Si-ming Xu, Ke Lu, Xu-feng Yang, Yao-wei Ye, Min-zhe Xu, Qin Shi, Ya-qin Gong, Chong Li. "Association of 25-hydroxyvitamin D levels wit...
CROSSREF
<1%

20 findresearcher.sdu.dk
INTERNET
<1%

21 "30th Annual San Antonio Breast Cancer Symposium – December 13–16, 2007", Breast Cancer Research and Treatment, 2007
CROSSREF
<1%

22 bmcpsychiatry.biomedcentral.com
INTERNET
<1%

23 T. Cigler, H. Richardson, M. J. Yaffe, C. J. Fabian et al. "A randomized, placebo-controlled trial (NCIC CTG MAP.2) examining the effects ...
CROSSREF
<1%

24 ojrd.biomedcentral.com
INTERNET
<1%

25 Ganggang Wang, Zhijie Zhou, Wenzhi Jin, Xin Zhang, Hao Zhang, Xiaoliang Wang. "Single-cell transcriptome sequencing reveals spatial...
CROSSREF
<1%

26 Kaiyue Wang, Lu Shen, Wei Tian, Suzhan Zhang. "Comparison of changes in lipid profiles of premenopausal women with early-stage br...
CROSSREF
<1%

27 Qing, Ying. "Correlation between Helicobacter pylori-associated gastric diseases and colorectal neoplasia", World Journal of Gastroent...
CROSSREF
<1%

28 synapse.koreamed.org
INTERNET
<1%

29 scholarbank.nus.edu.sg
INTERNET
<1%

30 www.100md.com
INTERNET
<1%

31 www.doria.fi
INTERNET
<1%

Preprint source
32 Luyao Wang, Yifan Chu, Jiaxin Xie, Shulin Yang, Jing Yue. "Outcomes of fertility preservation before or after anticancer treatments in y...
CROSSREF POSTED CONTENT
<1%

33 pubmed.ncbi.nlm.nih.gov
INTERNET
<1%

34 www.hindawi.com
INTERNET
<1%

35 www.imrpress.com
INTERNET
<1%

36 Akiho Tamura. "Unfavorable lipid profiles in mild obesity with excess body fat percentage", Pediatrics International, 1/24/2000
CROSSREF
<1%

37 Muhammad Younus. "Putting the Cardiovascular Safety of Aromatase Inhibitors in Patients with Early Breast Cancer into Perspective ...
CROSSREF
<1%

38 Stéphanie Gaillard, Vered Stearns. "Aromatase inhibitor-associated bone and musculoskeletal effects: new evidence defining etiology a...
CROSSREF
<1%

39 Yongbo Liang, Zhencheng Chen, Rabab Ward, Mohamed Elgendi. "Photoplethysmography and Deep Learning: Enhancing Hypertensio...
CROSSREF
<1%

40 bmcbiotechnol.biomedcentral.com
INTERNET
<1%

41 journals.lww.com
INTERNET
<1%

42 journals.plos.org
INTERNET
<1%

43 Allison J. Carroll, Mark D. Huffman, Lihui Zhao, David R. Jacobs, Jesse C. Stewart, Catarina I. Kiefe, Kiang Liu, Brian Hitsman. "Evaluati...
CROSSREF
<1%

44 Hongwei Li, Runlu Sun, Qian Chen, Qi Guo, Junjie Wang, Liming Lu, Yuling Zhang. "Association between HDL-C levels and menopause: ...
CROSSREF
<1%

45 Kasat, Shilpa S.. "Effect of Hemodialysis in Patients with Chronic Renal Failure on Lipid Profile, Serum Paraoxonase-1 Activity and Mal...
PUBLICATION
<1%

46 Matthias Blüher. "INFLUENCE OF DIETARY INTAKE AND PHYSICAL ACTIVITY ON ANNUAL RHYTHM OF HUMAN BLOOD CHOLESTERO...
CROSSREF
<1%

47 Valentino Martelli, Maria Maddalena Latocca, Tommaso Ruelle, Marta Perachino et al. "Comparing the Gonadotoxicity of Multiple Brea...
CROSSREF
<1%

48 Xiaoli Liao, Yue Li, Zhenzhu Hu, Ying Lin, Bo Zheng, Jihua Ding. "Poplar acetylome profiling reveals lysine acetylation dynamics in seaso...
CROSSREF
<1%

49 Yanmin Liu, Zhihao Dang, Yanhui Wang, Megha N. Parajulee, Fajun Chen. "Interactive Effects of [CO2] and Temperature on Plant Chem...
CROSSREF
<1%

50 cardiab.biomedcentral.com
INTERNET
<1%

51 dspace.plymouth.ac.uk
INTERNET
<1%

52 jamanetwork.com
INTERNET
<1%

53 static-site-aging-prod2.impactaging.com
INTERNET
<1%

54 unsworks.unsw.edu.au
INTERNET
<1%

55 worldwidescience.org
INTERNET
<1%

56 xuebao.jlu.edu.cn
INTERNET
<1%

57 Bond-Bero, Stacy. "Filling the Gap for Early-Stage Breast Cancer Follow-Up: An Overview for Primary Care Providers", Journal of Midwif...
CROSSREF
<1%

58 Breast Disease, 2016.
CROSSREF
<1%

59 Tao He, Wenhao Yang, Xinyi Zhang, Ping Li et al. "Comparative effectiveness of tamoxifen, toremifene, letrozole, anastrozole, and exem...
CROSSREF
<1%

60 Thomas Jr, Debabrata Mukherjee. "Medications not Intended for Treatment of Dyslipidemias and with a Variable Effect on Lipids", Curr...
CROSSREF
<1%

61 "ORALS", Journal Of Clinical Periodontology, 7/2006
CROSSREF
<1%

62 Christos J Markopoulos, Alexandra K Tsaroucha, Helen J Gogas. "Effect of aromatase inhibitors on the lipid profile of postmenopausal...
CROSSREF
<1%

63 Shuwen Dong, Zheng Wang, Kunwei Shen, Xiaosong Chen. "Metabolic Syndrome and Breast Cancer: Prevalence, Treatment Response, ...
CROSSREF
<1%

64 Yuechong Li, Yingjiao Wang, Qiang Sun, Songjie Shen. "Clinicopathologic features, treatment, and prognosis of pregnancy-associated ...
CROSSREF
<1%

Excluded search repositories:

- None

Excluded from document:

- Bibliography

Excluded sources:

- None

Excluded preprints

- None

Lipid changes during endocrine therapy in early-stage breast cancer patients: A real-world study

Yuechong Li[1†], Zixi Deng[1†], Yingjiao Wang[1], Songjie Shen[1*]

[1] Department of Breast Surgery, Peking Union Medical College Hospital, Beijing, China

[†] Yuechong Li and Zixi Deng contributed equally to this work.

*CORRESPONDENCE
Songjie Shen
pumcssj@163.com

Abstract

Background: Endocrine drugs may affect lipid metabolism in breast cancer (BC) patients. This study aims to explore the lipid changes in early-stage BC patients taking different endocrine drugs.

Methods: The change trend of blood lipid during endocrine therapy in 2756 BC patients from January 2013 to December 2021 were retrospectively analyzed. The changes of four lipid parameters were assessed by the Generalized Linear Mixed Model, including total cholesterol (TC), triglycerides (TG), low-density lipoprotein (LDL-C), and high-density lipoprotein (HDL-C). These parameters were quantified at baseline and at 6, 12, 18, 24, 36, 48, 60, and 72 months after initiation of endocrine therapy. Subgroup analysis according to menopausal status or medication types was conducted.

Results: A total of 1201 patients taking aromatase inhibitors (AIs), including anastrozole (ANA), letrozole (LET) or exemestane (EXE), and 1555 patients taking toremifene (TOR) were enrolled. TC and TG levels showed a significantly elevated trend during 5 years of treatment ($P<0.05$). HDL-C levels increased from baseline in the TOR group ($P<0.05$). Compared with the postmenopausal AI group, the increasing trends of TC, TG, and LDL-C in the premenopausal AI group were more evident with the extension of time (β=0.105, 0.027, 0.086, respectively). Within 3 years, TC, TG, and LDL-C levels in the ANA and LET groups were significantly higher than baseline levels ($P<0.05$). Furthermore, the levels of TG in the EXE group were significantly lower than that in the ANA or LET group ($P<0.05$), but this significant difference disappeared after 3 years.

Conclusions: AIs significantly influenced lipid profiles more than TOR. AIs had a greater effect on blood lipid in premenopausal patients. Steroidal AIs (EXE) may have a smaller effect on lipid levels than nonsteroidal AIs (ANA and LET).

Keywords: lipid profile; breast cancer; endocrine therapy; toremifene; aromatase inhibitors

Background

The latest global cancer burden data revealed that breast cancer (BC) has eclipsed lung cancer as the most predominant malignancy worldwide, accounting for nearly

2.26 million newly diagnosed cases globally[1]. Notably, the incidence of BC is also increasing annually in China[2]. BC with hormone receptors (HR) positive is the most common subtype, constituting approximately 60% of all cases[3], deserving urgent exploration.

Adjuvant endocrine therapy is an essential component of comprehensive treatment for patients with HR-positive BC and lasts 5-10 years. Endocrine drugs include two main types, namely selective estrogen receptor modulators (SERMs) and aromatase inhibitors (AIs). Toremifene (TOR) is one type of SERMs. AIs mainly divided into nonsteroidal AIs, as anastrozole (ANA) and letrozole (LET), and steroidal AIs as exemestane (EXE). They can inhibit the growth of BC through competitive binding with estrogen receptors or reduce estrogen levels by suppressing aromatase activity. In doing so, these therapies substantially improve the prognosis of HR-positive BC with lower recurrence rates and better overall survival[4]. However, estrogen plays a variety of physiological functions, including lipid and bone metabolism and cardiovascular, cognitive, and sexual functions[5]. Studies have also demonstrated that prolonged diminution of estrogen levels over an extended period may precipitate dyslipidemia, thereby elevating the susceptibility to cardiovascular diseases (CVD), such as myocardial infarction and stroke[6-8]. They may be even more noticeable in premenopausal BC survivors due to the abrupt suppression of estrogen[9]. In particular, CVD is estimated to be the leading cause of noncancer deaths in BC patients, especially for elderly people with early-stage BC[10]. Therefore, a

comprehensive investigation of the enduring side effects of endocrine therapy is imperative.

Little consensus existed regarding the specific role of endocrine drugs in lipid metabolism. Some studies claimed that toremifene (TOR) is associated with a favorable influence on lipid profiles, with reduced triglyceride (TG) and increased high-density lipoprotein cholesterol (HDL-C)[11]. Other studies reported adverse lipid profile effects of endocrine therapy in premenopausal BC patients[12]. Additionally, a small-scale clinical trial conducted among postmenopausal Chinese BC patients indicated that nonsteroidal AIs increased the risk of lipid events[13]. Moreover, there are few studies on the impact of various endocrine drugs on lipid profiles, especially the comparisons between AIs in real-world studies. Comprehending alterations in blood lipid profiles during endocrine therapy and the impact of diverse endocrine drugs on blood lipid contributes to informed decision-making regarding endocrine drug selection for individual patients in clinical practice. Therefore, this large-scale, real-world retrospective study aims to investigate the blood lipid changes throughout endocrine therapy and assess the influence of diverse endocrine drugs on lipid metabolism.

Methods

Study population

Using the big data query and analysis system, 4886 BC patients undergoing endocrine

therapy were retrospectively enrolled between January 1, 2013 and December 31, 2021 at Peking Union Medical College Hospital (PUMCH). Ten male patients were excluded. Two hundred seventy-two patients who received multiple SERMs or AIs during endocrine therapy were excluded to avoid interference with the analysis due to alterations in endocrine drugs. Additionally, 1848 patients with dyslipidemia before the initiation of medication were excluded. Overall, 2756 stage I-III BC patients with endocrine therapy were enrolled. Figure 1 showed the patient selection flowchart. This study was approved by the Ethics Committee of PUMCH (approval number: I-22PJ227). All patients signed informed consent for related treatment. The inclusion criteria comprised the following: (1) ≥18 year-old female patients; (2) stage I-III BC patients; (3) patients with HR-positive BC who have completed endocrine therapy for at least 6 months; (4) patients who received letrozole (LET), anastrozole (ANA), exemestane (EXE), or TOR as endocrine therapy. The exclusion criteria comprised the following: (1) patients who changed endocrine drugs, namely patients who have taken more than one endocrine drug; (2) patients with dyslipidemia or patients who took lipid-lowering drugs before endocrine therapy; (3) patients who received endocrine therapy during neoadjuvant therapy or before BC diagnosis. All enrolled patients received surgical procedures and systemic therapies per the National Comprehensive Cancer Network (NCCN) guidelines. All premenopausal women who received AIs were injected with ovarian function suppression (OFS) drugs. Dyslipidemia was defined as meeting any of the following criteria: total cholesterol (TC)≥5.2 mmol/L, TG≥1.7 mmol/L, low-density lipoprotein cholesterol (LDL-C)≥

3.4 mmol/L, and HDL-C<1.0 mmol/L[14].

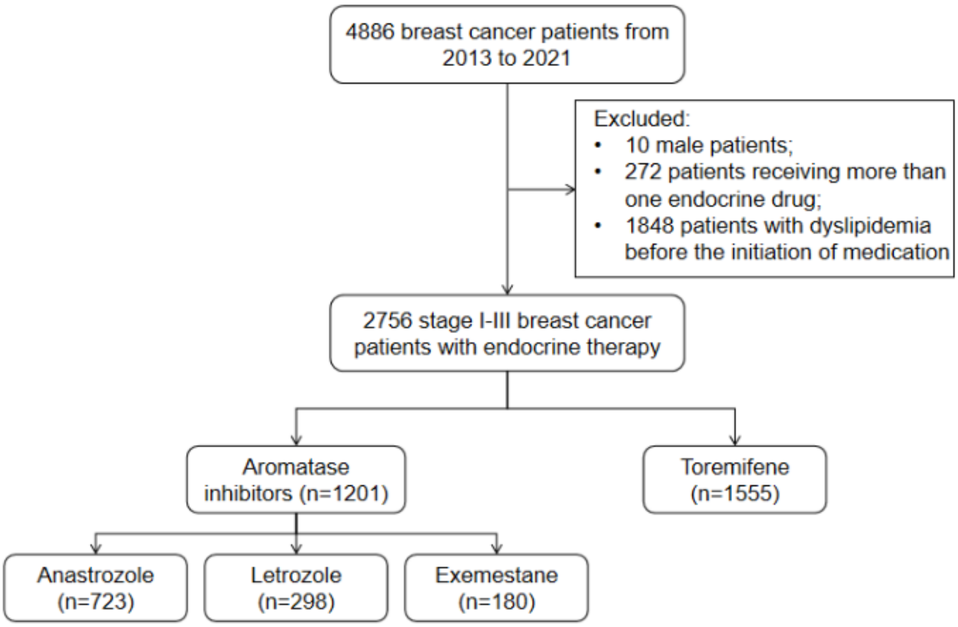

Figure 1. Patient selection flowchart.

Data collection

Data collected included age at diagnosis, height, weight, body mass index (BMI), menopausal status, treatment (breast-conserving surgery or mastectomy, chemotherapy, endocrine therapy, and targeted therapy), comorbidities (hypertension, coronary heart diseases, diabetes, and fatty liver), and lipid levels at baseline and at 6, 12, 18, 24, 36, 48, 60 and 72 months after initiation of endocrine therapy. BMI was calculated as the weight divided by the square of height (Kg/m^2). Furthermore, lipid levels include TC, TG, LDL-C, and HDL-C.

Statistical analysis

Statistical comparisons of baseline lipid profiles and covariates among various subgroups, as applicable, were conducted using Pearson's chi-square, Fisher's exact, Student's t-tests, or Analysis of Variance (ANOVA) tests.

The Generalized Linear Mixed Model was employed to assess alterations in lipid profiles across various endocrine therapy groups at different administration time points. Model-adjusted least-square means were used to describe the blood lipid levels in different subgroups. All tests were two-sided. $P<0.05$ was considered statistically significant. Statistical analyses were performed using SPSS 25.0 statistical software (IBM Corporation, Armonk, NY, USA).

Results

Comparison of BC patients taking AIs or TOR

A total of 2756 patients were included: 1201 patients taking AIs and 1555 patients taking TOR. The baseline characteristics are shown in Table 1. The average age and BMI in the AI group were higher than those in the TOR group ($P<0.001$). Furthermore, the proportion of patients in the TOR group undergoing breast-preserving surgery was higher than that of the AI group ($P<0.001$). In the AI group, The baseline lipid profiles is higher than the TOR group ($P<0.001$).

Table 1. Baseline characteristics of BC patients taking AIs or TOR.

Variables	AIs (n=1201)	TOR (n=1555)	$P^{\#}$
Age, mean（SD）	55.61 (11.89)	42.2 (6.71)	<0.001
BMI（SD）	24.18 (3.65)	22.93 (3.42)	<0.001
Surgery			<0.001
Breast conserving	486 (40.5)	762 (49.0)	
Mastectomy	680 (56.6)	736 (47.3)	
Adjuvant therapy			
Target therapy	202 (16.8)	139 (8.9)	<0.001
Chemotherapy	542 (45.1)	430 (27.7)	<0.001
Comorbidities			
Hypertension	179 (14.9)	233 (15.0)	0.954
Coronary diseases	14 (1.2)	24 (1.5)	0.399
Diabetes	51 (4.2)	83 (5.3)	0.187

Fatty liver	1 (0.1)	5 (0.3)	0.241
Baseline lipid profiles			
TC, mean (SD)	4.41 (0.53)	4.37 (0.50)	0.132
TG, mean (SD)	0.95 (0.34)	0.81 (0.32)	<0.001
LDL-C, mean (SD)	2.46 (0.51)	2.44 (0.46)	0.352
HDL-C, mean (SD)	1.42 (0.25)	1.43 (0.25)	0.122

[#] Comparisons of surgery type, adjuvant therapy and comorbidities are conducted using Pearson's chi-square or Fisher's exact; comparisons of age, BMI and baseline lipid profiles are conducted using Student's t-tests.

The changing trend of different lipid indexes in each group over time is shown in Figure 2. Compared with the baseline of each group, TC and TG levels were significantly higher at 6 months after taking the drug (P<0.05) and the upward trend existed during the subsequent 5 years of treatment (P<0.05). Compared with baseline, LDL-C levels in the AI group significantly increased (P<0.05), while that in the TOR group showed an increasing trend with no significant difference. HDL-C levels in the AI group significantly decreased from baseline levels at 6 months and 1 year after medication (P<0.05), and those in the TOR group increased from baseline levels (P<0.05).

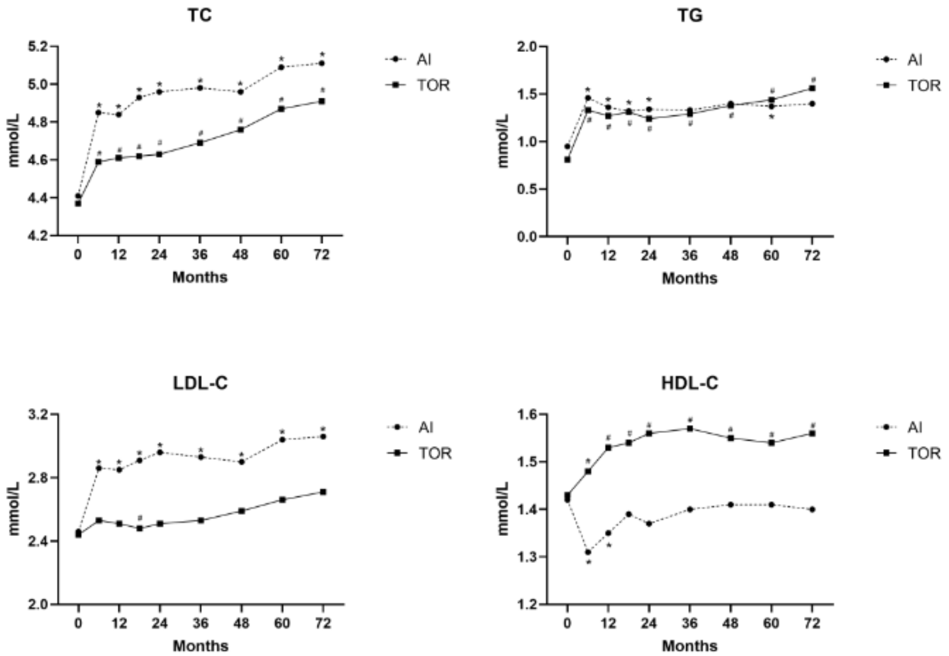

Figure 2. Changes of lipid profiles in AIs and TOR groups compared with baseline using Student's t-tests; *represents $P<0.05$ in the AI group; #represents $P<0.05$ in the TOR group.

In general, TC, TG, and LDL-C levels showed an increasing trend with the extension of medication time (β=0.063, 0.054, 0.057, respectively) (Table 2). Compared with the AI group, TC and LDL-C levels in the TOR group increased less significantly with the extension of time (β=-0.020, -0.055, respectively). Furthermore, TC and TG levels exhibited positive associations with age (β=0.005, 0.006, respectively), and TG and LDL-C levels exhibited positive associations with BMI (β=0.031, 0.017, respectively). In contrast, HDL-C levels exhibited negative associations BMI (β=-0.027).

Table 2. Comparison of lipid profiles in BC patients taking AIs or TOR.

	TC (β[#], 95%CI)	TG (β[#], 95%CI)	LDL-C (β[#], 95%CI)	HDL-C (β[#], 95%CI)
Endocrine therapy				
AIs	0	0	0	0
TOR	-0.015 (-0.118,0.089)	-0.034 (-0.101, 0.033)	0.008 (-0.083,0.099)	0.034 (-0.006,0.074)
Time	0.063 (0.052,0.074)	0.054 (0.043,0.064)	0.057 (0.047,0.067)	0.009 (0.005,0.012)
Time*endocrine therapy				
AIs*time	0	0	0	0
TOR*time	-0.020 (-0.035, -0.005)	0.033 (0.018,0.047)	-0.055 (-0.069, -0.042)	0.020 (0.014,0.025)
Age	0.005 (0.001,0.009)	0.006 (0.004,0.009)	0.001 (-0.002,0.005)	0.001 (-0.001,0.002)
BMI	-0.005 (-0.015,0.006)	0.031 (0.024,0.039)	0.017 (0.008,0.026)	-0.027 (-0.031, -0.023)
Surgery				
Breast conserving	0	0	0	0
Mastectomy	0.167 (0.096,0.237)	0.068 (0.019,0.117)	0.151 (0.088,0.213)	-0.030 (-0.058, -0.003)
Adjuvant therapy				
Target therapy	0.079 (-0.040,0.198)	-0.001 (-0.082,0.081)	0.076 (-0.029, 0.182)	-0.001 (-0.048,0.045)
Chemotherapy	-0.007 (-0.093,0.079)	-0.055 (-0.114,0.004)	0.006 (-0.070,0.082)	0.024 (-0.010,0.058)
Comorbidities				
Hypertension	-0.012 (-0.110,0.087)	-0.007 (-0.075,0.061)	0.019 (-0.068,0.106)	-0.006 (-0.045,0.033)

[#] Generalize Linear Mixed Models are used; the time of administration was taken as a continuous variable.

Subsequently, the lipid profiles at each assessment time point were compared (Supplementary Table 1). In the AI group, the levels of LDL-C were significantly higher than the TOR group during 5 years of endocrine therapy ($P<0.05$). TC and LDL-C levels in the AI group were significantly higher than those in the TOR group from 18 months to 3 years after medication ($P<0.05$). Furthermore, the levels of

HDL-C in the TOR group were significantly higher than those in the AI group during the medication time ($P<0.05$).

Comparison of premenopausal and postmenopausal patients taking AIs

Next, subgroup analysis was conducted for patients taking AIs according to menopause status. The baseline characteristics are shown in Supplementary Table 2. Of the 1201 patients taking AIs, 889 were postmenopausal, and 312 were premenopausal. Notably, postmenopausal patients' mean age and BMI were higher than premenopausal patients ($P<0.001$).

Compared with the baseline lipid profiles, the levels of TC and LDL-C in each group showed a significant increasing trend ($P<0.05$) (Figure 3). The TG levels in the two groups peaked at 6 months after receiving therapy, and were significantly higher than baseline ($P<0.05$). At 12, 18, 24, 36, 60 months after medication, TG levels were still higher in the two groups than baseline ($P<0.05$). Moreover, the HDL-C levels in both groups decreased significantly at 6 months after medication ($P<0.05$). After 12 months of medication, the HDL-C levels in both groups are not significantly different from baseline levels ($P>0.05$).

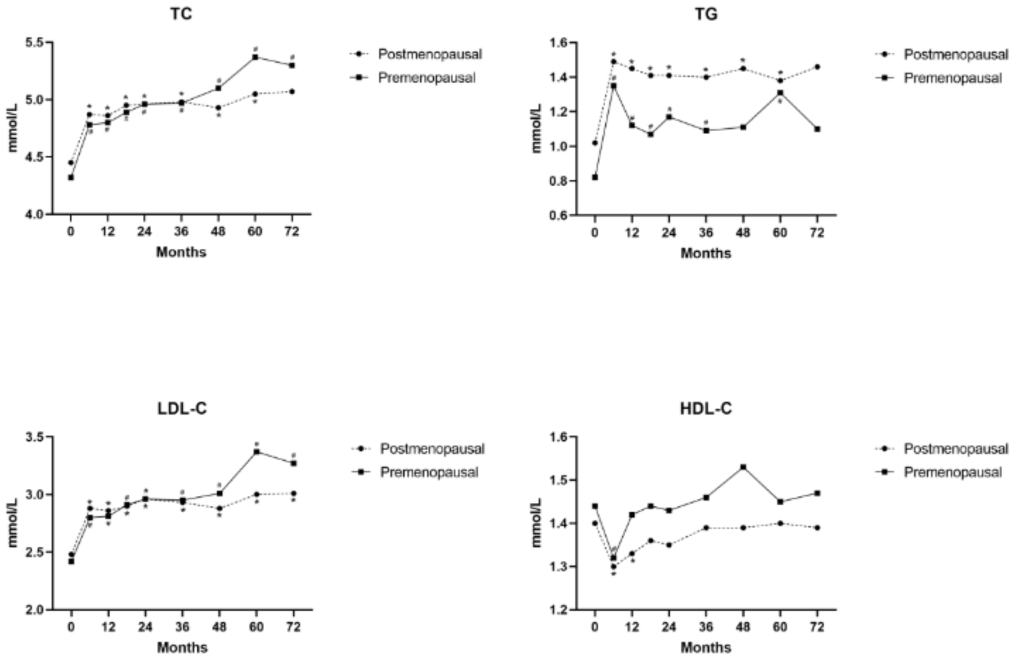

Figure 3. Changes of lipid profiles in premenopausal and postmenopausal groups compared with baseline using Student's t-tests; * represents $P<0.05$ in the postmenopausal group; # represents $P<0.05$ in the premenopausal group.

Compared with the postmenopausal AI group, the increasing trends of TC, TG, and LDL-C in the premenopausal AI group were more evident with the extension of time (β=0.105, 0.027, 0.086, respectively) (Table 3). Similarly, TG levels exhibited positive associations with BMI (β=0.030), and TC and HDL-C levels exhibited negative associations with BMI (β=-0.019, -0.023, respectively). Compared with the postmenopausal AI group, the levels of TC and LDL-C in the premenopausal AI group were lower within 1.5 years after medication ($P<0.05$) (Supplementary Table 3).

Table 3. Comparison of blood lipids in premenopausal and postmenopausal patients

taking AIs.

	TC ($\beta^{\#}$, 95%CI)	TG ($\beta^{\#}$, 95%CI)	LDL-C ($\beta^{\#}$, 95%CI)	HDL-C ($\beta^{\#}$, 95%CI)
Menopausal status				
postmenopausal	0	0	0	0
premenopausal	-0.721 (-0.921, -0.522)	-0.264 (-0.394, -0.134)	-0.611 (-0.787, -0.435)	0.012 (-0.063,0.087)
Time	0.037 (0.024,0.051)	0.032 (0.022,0.041)	0.034 (0.023,0.046)	0.009 (0.005,0.014)
Time*menopausal status				
Postmenopausal*time	0	0	0	0
Premenopausal*time	0.105 (0.079,0.130)	0.027 (0.009,0.045)	0.086 (0.064, 0.108)	0.002 (-0.006,0.010)
Age	-0.009 (-0.016, -0.003)	0.001 (-0.004,0.005)	-0.011 (-0.017, -0.005)	-0.001 (-0.003,0.002)
BMI	-0.019 (-0.034, -0.005)	0.030 (0.020,0.040)	-0.001 (-0.013,0.012)	-0.023 (-0.028, -0.017)
Surgery				
Breast conserving	0	0	0	0
Mastectomy	0.181 (0.075,0.287)	0.095 (0.023,0.167)	0.162 (0.068,0.256)	-0.037 (-0.077,0.002)
Adjuvant therapy				
Target therapy	0.034 (-0.125,0.192)	0.007 (-0.099,0.114)	-0.006 (-0.146,0.134)	0.010 (-0.049,0.069)
Chemotherapy	0.071 (-0.059,0.201)	-0.026 (-0.114,0.063)	0.118 (0.003,0.234)	-0.014 (-0.062,0.035)
Complication				
Hypertension	-0.061 (-0.208,0.086)	-0.037 (-0.136,0.063)	-0.033 (-0.164,0.097)	-0.001 (-0.055,0.055)

Generalize Linear Mixed Models are used; the time of administration was taken as a continuous variable.

Comparison of BC patients taking different AIs

Among 1201 patients taking AIs drugs, 723 patients received ANA treatment, 298 received LET treatment, and 180 received EXE treatment. The baseline characteristics are shown in Supplementary Table 4. The changing trend of lipid profiles in each

group is shown in Figure 4. During the 3 years after taking the drugs, the TC, TG, and LDL-C levels in the ANA and LET groups were significantly higher than baseline levels ($P<0.05$). The levels of TC and TG in the EXE group were not significantly different from the baseline level while the levels of LDL-C in the EXE group were significantly higher than baseline levels ($P<0.05$). At 6 months after taking the drugs, HDL-C levels in all groups were significantly lower than baseline levels ($P<0.05$). After that, HDL-C levels in the ANA and LET groups were not significantly different from baseline, with the curve gradually flattened. At 12, 36, 48 months after taking EXE, the HDL-C levels were significantly lower than baseline levels ($P<0.05$).

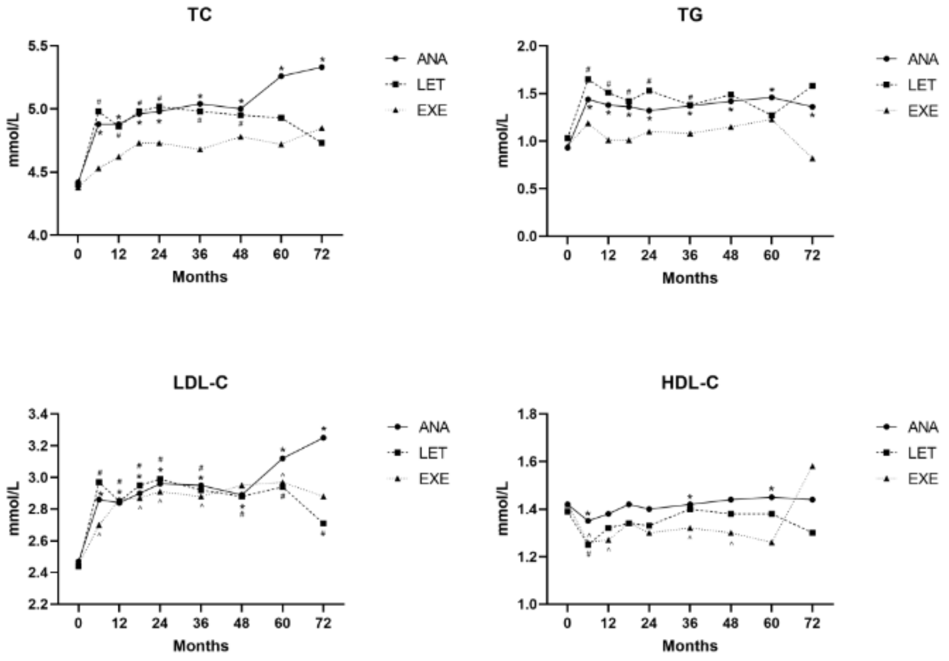

Figure 4. Changes of lipid profiles among different AIs groups compared with baseline using Student's t-tests; * represents $P<0.05$ in the ANA group; # represents $P<0.05$ in the LET group; ^ represents $P<0.05$ in the EXE group.

With the extension of time, the TC, TG, and LDL-C levels in different AIs groups showed an increasing trend (Supplementary Table 5). Additionally, BMI was positively correlated with TG (β=0.030) but was negatively correlated with HDL-C and TC levels (β=-0.019, -0.023, respectively).

Overall, the main difference in the effects of different AIs on lipid levels was between EXE and nonsteroidal AIs (Table 4). The TG level in the EXE group was lower within 3 years after medication than in the steroidal AIs groups (*P*<0.05). In contrast with the nonsteroidal AI groups, the levels of TC were lower in the EXE group at 12, 18, 36, and 48 months after medication (*P*< 0.05). Furthermore, compared with the ANA group, the levels of HDL-C were lower in the EXE group from 12 months to 60 months after medication (*P*<0.05).

Table 4. Comparison of blood lipids in BC patients taking different AIs at each medication time.

Months	ANA[&] (n=723)	LET[&] (n=298)	EXE[&] (n=180)	ANA vs LET $P^{\#}$	ANA vs EXE $P^{\#}$	LET vs EXE $P^{\#}$
TC						
6	4.87 (0.08)	4.96 (0.11)	4.65 (0.14)	0.477	0.140	0.066
12	4.86 (0.07)	4.80 (0.10)	4.40 (0.12)	0.566	<0.001	0.007
18	4.87 (0.07)	4.97 (0.10)	4.55 (0.12)	0.373	0.009	0.004
24	4.98 (0.06)	4.85 (0.09)	4.61 (0.12)	0.178	0.003	0.084
36	5.03 (0.07)	4.94 (0.10)	4.59 (0.14)	0.400	0.002	0.029
48	4.91 (0.08)	4.82 (0.11)	4.42 (0.15)	0.468	0.003	0.023
60	5.08 (0.11)	4.79 (0.13)	4.69 (0.22)	0.072	0.113	0.707
72	5.03 (0.15)	4.77 (0.17)	4.55 (0.41)	0.244	0.266	0.617
TG						
6	1.42 (0.06)	1.56 (0.09)	1.05 (0.10)	0.153	0.001	<0.001

12	1.37 (0.05)	1.40 (0.08)	0.91 (0.10)	0.752	<0.001	<0.001
18	1.35 (0.06)	1.35 (0.09)	1.01 (0.10)	0.964	0.002	0.008
24	1.26 (0.05)	1.37 (0.08)	1.00 (0.10)	0.212	0.015	0.002
36	1.33 (0.05)	1.26 (0.08)	0.98 (0.11)	0.448	0.004	0.038
48	1.24 (0.06)	1.19 (0.08)	1.02 (0.11)	0.614	0.085	0.212
60	1.26 (0.07)	1.18 (0.09)	1.10 (0.15)	0.484	0.351	0.656
72	1.42 (0.13)	0.97 (0.15)	0.89 (0.34)	0.017	0.141	0.815
LDL-C						
6	2.86 (0.07)	2.94 (0.10)	2.78 (0.12)	0.470	0.554	0.287
12	2.80 (0.06)	2.82 (0.09)	2.67 (0.10)	0.904	0.225	0.256
18	2.81 (0.06)	2.95 (0.09)	2.77 (0.10)	0.142	0.728	0.158
24	2.92 (0.06)	2.87 (0.08)	2.80 (0.11)	0.581	0.264	0.536
36	2.94 (0.06)	2.91 (0.09)	2.79 (0.12)	0.810	0.243	0.374
48	2.85 (0.07)	2.84 (0.09)	2.61 (0.13)	0.936	0.085	0.124
60	2.96 (0.10)	2.82 (0.12)	2.86 (0.20)	0.343	0.644	0.873
72	2.87 (0.13)	2.91 (0.15)	2.73 (0.36)	0.849	0.694	0.630
HDL-C						
6	1.33 (0.02)	1.27 (0.03)	1.32 (0.04)	0.056	0.796	0.230
12	1.39 (0.02)	1.31 (0.03)	1.26 (0.04)	0.022	0.001	0.208
18	1.40 (0.02)	1.34 (0.03)	1.29 (0.04)	0.041	0.002	0.235
24	1.44 (0.02)	1.35 (0.03)	1.34 (0.04)	0.004	0.007	0.702
36	1.46 (0.02)	1.40 (0.03)	1.33 (0.04)	0.077	0.003	0.140
48	1.47 (0.02)	1.40 (0.03)	1.31 (0.04)	0.024	<0.001	0.051
60	1.50 (0.03)	1.42 (0.04)	1.33 (0.06)	0.056	0.004	0.149
72	1.46 (0.04)	1.38 (0.05)	1.38 (0.11)	0.211	0.471	0.955

Generalize Linear Mixed Models are used to compare the blood lipids of different endocrine agents at each time point taking time of medication as a classified variable.

& Serum lipid levels were presented as model adjusted least-square means.

Finally, the proportion of dyslipidemia in each group was compared (Figure 5). During the five years of endocrine therapy, the proportion of dyslipidemia in the AI group was significantly higher than in the TOR group (P<0.01). One year after the initiation of medication, there was a higher proportion of dyslipidemia in the postmenopausal group than in the premenopausal group (P<0.01). Moreover, there was a lower proportion of dyslipidemia in those taking EXE than those in nonsteroidal AI groups (P<0.05).

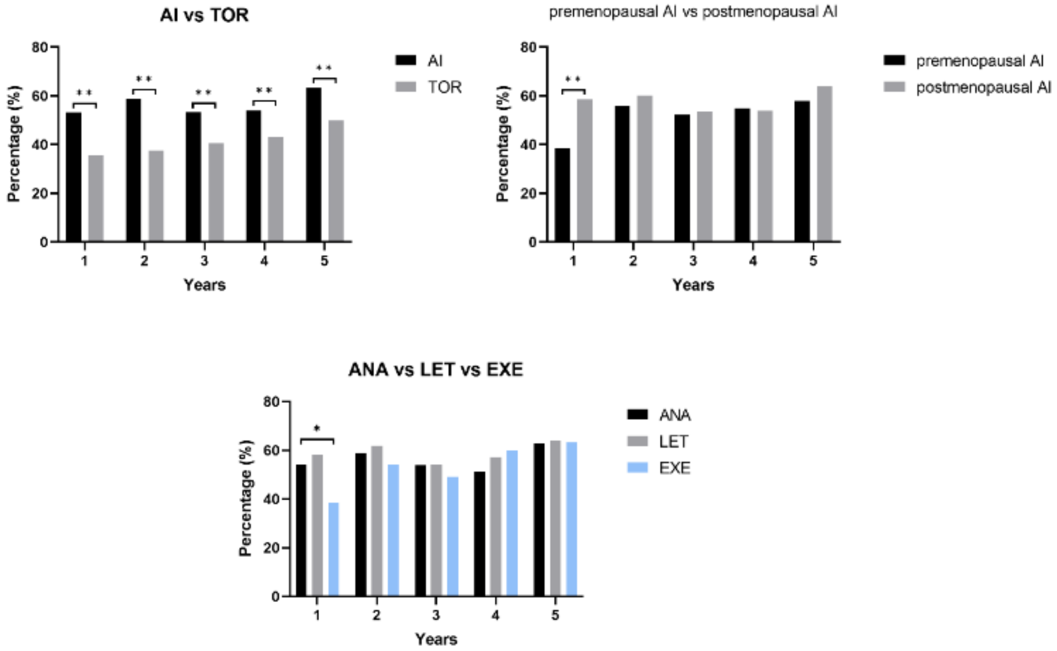

Figure 5. Comparison of the proportion of dyslipidemia in different groups using Pearson's chi-square. ** represents P<0.01, * represents P<0.05.

Discussion

In this real-world study in China, with long follow-up time and a large sample size, TOR exhibited favorable effects on HDL-C levels, while AIs negatively influenced TC, TG, and LDL-C levels. For subgroup analysis, AIs may significantly affect lipid profiles in both premenopausal and postmenopausal BC patients, with a more evident increasing trend of TC, TG and LDL-C in the premenopausal AI group. EXE tended to have a minor effect on the levels of TC and TG than ANA and LET.

TOR, another type of SERM, has demonstrated comparable efficacy to tamoxifen (TAM) in HR-positive BC patients[15, 16]. It is widely used in China, so this study

explored its effects on lipid profiles. The previous findings regarding the effects of TAM on blood lipid remain unclear. A randomized controlled study showed TAM lowered LDL-C and TC levels but with a small sample size and short follow-up time[17]. Other research demonstrated that TAM increased TG and LDL-C levels and was associated with more severe fatty liver disease and liver fibrosis[11, 18]. Song D et al. found that TOR improved the lipid profiles of premenopausal BC patients[11]. In this study, compared with baseline, the levels of TC and TG showed a significant increasing trend. LDL-C levels in the TOR group also showed an upward trend but no significant difference. Simultaneously, HDL-C levels increased in the TOR group. Therefore, TOR may have a protective effect on HDL-C levels, but it may still adversely affect the levels of TC and TG.

Regarding comparing the influences of AIs and TOR on lipid profiles, AIs negatively affected TC, TG, and LDL-C levels, which tended to have a greater impact on lipid profiles than TOR. These findings are consistent with previous studies. A randomized study demonstrated that the levels of TC and LDL-C were lower after TOR treatment than after LET treatment in a prospective clinical trial[19]. A 100-month follow-up of the ATAC trial revealed a higher incidence of hypercholesterolemia in the ANA group compared to the TAM group among postmenopausal BC patients[20]. A large cohort study also found that, whether postmenopausal or premenopausal, TC and LDL-C levels in the AI group were higher than the SERM group[21]. This difference may be due to the different mechanisms between AIs and SERMs. AIs inhibit estrogen

synthesis by reducing systemic aromatization, thus weakening the favorable effects of estrogen in lipid metabolism[22]. Moreover, the structure of SERM is similar to that of estrogen, which competes with estradiol to form a stable complex with estrogen receptors. As a result, SERM can perform an estrogenic function with a positive effect on blood lipids to some extent[23]. Previous studies have demonstrated that high TG levels and low HDL-C levels are important risk factors for CVD[24, 25]. Therefore, for patients at high risk of CVD, TOR may be a preferred option. It is necessary to closely monitor lipid profiles during endocrine therapy, especially for those patients taking AIs. However, there lack large-scale prospective studies regarding whether TOR has a protective effect on lipid metabolism, which needs further exploration.

As the increasing trends of TC, TG, and LDL-C levels in the premenopausal AI group were more apparent with the extension of time, premenopausal patients should pay more attention to blood lipid changes and clinicians need to guide the management of blood lipids during patients' follow-up visits. The abrupt suppression of estrogen in premenopausal patients may be the main reason. Research has indicated that using OFS and younger age may exacerbate lipid-related events in premenopausal patients[26]. However, a 5-year cohort study revealed a higher incidence of dyslipidemia in postmenopausal patients compared to their premenopausal counterparts (42.6% vs. 32.6%)[21]. This study also showed a higher incidence of dyslipidemia in postmenopausal patients especially at the first year after medication then the difference disappeared. This may be related to the higher baseline levels of

blood lipid in postmenopausal patients, which makes postmenopausal patients more prone to dyslipidemia. The use of lipid-lowering drugs may also affect the proportion of dyslipidemia. This finding can also likely be explained by other independent risk factors for female dyslipidemia, like BMI and age[27]. These factors may interact with each other and be associated with poor prognosis of BC. A recent study showed that obesity was associated with an increased risk of breast cancer recurrence among postmenopausal BC patients treated with AIs[28]. Studies also showed that dyslipidemia were associated with adverse outcomes of BC patients treated with endocrine therapy[29, 30].

Regarding the comparison of different AIs on lipid profiles, EXE appeared to have a smaller effect on lipid levels than nonsteroidal AIs (ANA and LET). This result is in accordance with previous studies. The MA.27 study showed that the increase of TG and TC levels was more prevalent in the ANA group than in the EXE group[31]. Wang et al. also demonstrated that compared with steroidal AI group, nonsteroidal AI group developed a higher cumulative incidence of dyslipidemia[13]. In this study, the levels of HDL-C in EXE group decreased significantly. This is in accordance with a small sample-size study which showed that HDL-C levels in EXE group decreased significantly than placebo group at 3, 6, 12 months[32]. A possible reason for that may be the metabolite of EXE, 17-hydroxy exemestane, which enhances the efficacy of suppressing aromatase[33]. Nevertheless, a prospective study in Japan showed that EXE and nonsteroidal AIs did not significantly affect lipid profiles[32, 34]. This study

further showed that such differences between steroidal and nonsteroidal AIs may disappear 3 years after medication. There may be a delayed effect of lipid changes for EXE，a two-year cohort study[32] showed that EXE group displayed no significant differences in percent change in LDL or TG at any time point compared with placebo group. The use of lipid-lowering drugs may also play a role. Thus, systematic reviews and further large-scale prospective trials are warranted[35].

Actually, for subgroup analysis of patients taking AIs, significant changes of TG and HDL-C levels could be seen at almost 6 months after taking the drug. However, since then, the degree of changes in these two parameters decreased a bit. This phenomenon may be related to the prescription of lipid-lowering drugs or the clinical doctors' advice to control lipid levels by changing lifestyle after the first follow-up of dyslipidemia. Studies have shown that lifestyle changes can help people control their lipid levels[36]. From this point of view, apart from the effects of lipid-lowering drugs, the conclusions of this study may also enlighten us to put the adjustment of lifestyle to control blood lipid levels in the postoperative instruction, which will be more beneficial to the control of blood lipid in the entire endocrine treatment.

Study strengths and limitations

Different endocrine drugs exhibit varying impacts on blood lipid profiles. Therefore, in the clinical treatment of women with dyslipidemia or elevated cardiovascular risk, preference should be given to endocrine drugs with minimal effects on blood lipids. This large-scale, real-world study systematically investigated changing trends in

blood lipid profiles during endocrine therapy and conducted comparative analyses of the influence of diverse endocrine drugs on blood lipids. Such insights are paramount for the judicious selection of endocrine therapies tailored to individual patients in clinical practice. For instance, while AIs generally demonstrate superior overall efficacy compared to SERM, this distinction diminishes for women at low risk of BC[37]. Considering SERM may be more appropriate in cases involving women at a high risk of cardiovascular disease. Among AIs, efficacy remains consistent, but distinctions emerge in their effects on lipid profiles. Moreover, patient comorbidities should inform AI selection decisions. There exist some limitations. This study is retrospective, single-center and lack of prognostic data regarding CVD, fatty liver, and other events. In addition, there is a lack of data on other factors influencing lipid levels, such as the use of lipid-lowering drugs, BMI changes during entire treatment, daily calorie intake, consumption, and lifestyle. The mechanism underlying the endocrine drugs affecting blood lipids is still unclear. Therefore, prospective randomized controlled trials on the influences of endocrine therapy on blood lipids and deeper exploration of how endocrine drugs affect blood lipids are needed.

Conclusion

In conclusion, compared with TOR, AIs tended to have a greater influence on lipid profiles. The increasing trends of TC, TG, and LDL-C levels were more evident in the premenopausal AI group, and EXE may have a minor effect on lipid levels than nonsteroidal AIs in the short term. Research into tools to assess endocrine drugs'

impacts on lipid profiles and comorbidities should be considered.

List of abbreviations

AI: Aromatase inhibitors

ANA: Anastrozole

ANOVA: Analysis of Variance

BC: Breast cancer

CVD: Cardiovascular diseases

EXE: Exemestane

HDL-C: High-density lipoprotein

HR: Hormone receptor

LDL-C: Low-density lipoprotein

LET: Letrozole

NCCN: National Comprehensive Cancer Network

OFS: Ovarian function suppression

PUMCH: Peking Union Medical College Hospital

SERM: Selective estrogen receptor modulators

TC: Total cholesterol

TG: Triglycerides

TOR: Toremifene

References

1. Sung H, Ferlay J, Siegel RL, Laversanne M, Soerjomataram I, Jemal A, et al. Global Cancer Statistics 2020: GLOBOCAN Estimates of Incidence and Mortality Worldwide for 36 Cancers in 185 Countries. CA Cancer J Clin. 2021;71(3):209-49.

2. Chen W, Zheng R, Baade PD, Zhang S, Zeng H, Bray F, et al. Cancer statistics in China, 2015. CA Cancer J Clin. 2016;66(2):115-32.
3. Roberto M, Astone A, Botticelli A, Carbognin L, Cassano A, D'Auria G, et al. CDK4/6 Inhibitor Treatments in Patients with Hormone Receptor Positive, Her2 Negative Advanced Breast Cancer: Potential Molecular Mechanisms, Clinical Implications and Future Perspectives. Cancers (Basel). 2021;13(2).
4. Dubsky P, Filipits M, Jakesz R, Rudas M, Singer CF, Greil R, et al. EndoPredict improves the prognostic classification derived from common clinical guidelines in ER-positive, HER2-negative early breast cancer. Ann Oncol. 2013;24(3):640-7.
5. Goss PE. Risks versus benefits in the clinical application of aromatase inhibitors. Endocr Relat Cancer. 1999;6(2):325-32.
6. Akyol M, Alacacioglu A, Demir L, Kucukzeybek Y, Yildiz Y, Gumus Z, et al. The alterations of serum FGF-21 levels, metabolic and body composition in early breast cancer patients receiving adjuvant endocrine therapy. Cancer Biomark. 2017;18(4):441-9.
7. Iorga A, Cunningham CM, Moazeni S, Ruffenach G, Umar S, Eghbali M. The protective role of estrogen and estrogen receptors in cardiovascular disease and the controversial use of estrogen therapy. Biol Sex Differ. 2017;8(1):33.
8. Schenck-Gustafsson K. Risk factors for cardiovascular disease in women: assessment and management. Eur Heart J. 1996;17 Suppl D:2-8.
9. Franzoi MA, Agostinetto E, Perachino M, Del Mastro L, de Azambuja E, Vaz-Luis I, et al. Evidence-based approaches for the management of side-effects of adjuvant endocrine therapy in patients with breast cancer. Lancet Oncol. 2021;22(7):e303-e13.
10. Patnaik JL, Byers T, DiGuiseppi C, Dabelea D, Denberg TD. Cardiovascular disease competes with breast cancer as the leading cause of death for older females diagnosed with breast cancer: a retrospective cohort study. Breast Cancer Res. 2011;13(3):R64.
11. Song D, Hu Y, Diao B, Miao R, Zhang B, Cai Y, et al. Effects of Tamoxifen vs. Toremifene on fatty liver development and lipid profiles in breast Cancer. BMC Cancer. 2021;21(1):798.
12. Cheung YM, Ramchand SK, Yeo B, Grossmann M. Cardiometabolic Effects of Endocrine Treatment of Estrogen Receptor-Positive Early Breast Cancer. J Endocr Soc. 2019;3(7):1283-301.
13. Wang X, Zhu A, Wang J, Ma F, Liu J, Fan Y, et al. Steroidal aromatase inhibitors have a more favorable effect on lipid profiles than nonsteroidal aromatase inhibitors in postmenopausal women with early breast cancer: a prospective cohort study. Ther Adv Med Oncol. 2020;12:1758835920925991.
14. Management JCotCGfL. Chinese guidelines for lipid management (2023). Zhonghua Xin Xue Guan Bing Za Zhi. 2023;51(3):221-55.
15. Pagani O, Gelber S, Price K, Zahrieh D, Gelber R, Simoncini E, et al. Toremifene and tamoxifen are equally effective for early-stage breast cancer: first results of International Breast Cancer Study Group Trials 12-93 and 14-93. Ann Oncol. 2004;15(12):1749-59.
16. Qin T, Yuan ZY, Peng RJ, Zeng YD, Shi YX, Teng XY, et al. Efficacy and tolerability of toremifene and tamoxifen therapy in premenopausal patients with operable breast cancer: a retrospective analysis. Curr Oncol. 2013;20(4):196-204.
17. Markopoulos C, Polychronis A, Dafni U, Koukouras D, Zobolas V, Tzorakoleftherakis E, et al. Lipid changes in breast cancer patients on exemestane treatment: final results of the TEAM Greek substudy. Ann Oncol. 2009;20(1):49-55.

18. Filippatos TD, Liberopoulos EN, Pavlidis N, Elisaf MS, Mikhailidis DP. Effects of hormonal treatment on lipids in patients with cancer. Cancer Treat Rev. 2009;35(2):175-84.
19. Shien T, Doihara H, Sato N, Anan K, Komaki K, Miyauchi K, et al. Serum lipid and bone metabolism effects of Toremifene vs. Letrozole as adjuvant therapy for postmenopausal early breast cancer patients: results of a multicenter open randomized study. Cancer Chemother Pharmacol. 2018;81(2):269-75.
20. Buzdar A, Howell A, Cuzick J, Wale C, Distler W, Hoctin-Boes G, et al. Comprehensive side-effect profile of anastrozole and tamoxifen as adjuvant treatment for early-stage breast cancer: long-term safety analysis of the ATAC trial. Lancet Oncol. 2006;7(8):633-43.
21. Wang J, Yin J, Qiu J, Jiang J, Hu Y, Zhu K, et al. Comparison of dyslipidemia incidence in Chinese early-stage breast cancer patients following different endocrine therapies: A population-based cohort study. Front Endocrinol (Lausanne). 2022;13:815960.
22. Stevenson JC, Crook D, Godsland IF. Influence of age and menopause on serum lipids and lipoproteins in healthy women. Atherosclerosis. 1993;98(1):83-90.
23. Wang K, Shen L, Tian W, Zhang S. Comparison of changes in lipid profiles of premenopausal women with early-stage breast cancer treated with different endocrine therapies. Sci Rep. 2022;12(1):22650.
24. Assmann G, Schulte H. Relation of high-density lipoprotein cholesterol and triglycerides to incidence of atherosclerotic coronary artery disease (the PROCAM experience). Prospective Cardiovascular Münster study. Am J Cardiol. 1992;70(7):733-7.
25. Manninen V, Tenkanen L, Koskinen P, Huttunen JK, Mänttäri M, Heinonen OP, et al. Joint effects of serum triglyceride and LDL cholesterol and HDL cholesterol concentrations on coronary heart disease risk in the Helsinki Heart Study. Implications for treatment. Circulation. 1992;85(1):37-45.
26. Kang YK, Wang X, Hu NL, Yue J, Si YR, Ju J, et al. The Effects of Endocrine Therapies on Lipid Profiles in Chinese Young Women With Early Breast Cancer. Front Oncol. 2021;11:759595.
27. Qi L, Ding X, Tang W, Li Q, Mao D, Wang Y. Prevalence and Risk Factors Associated with Dyslipidemia in Chongqing, China. Int J Environ Res Public Health. 2015;12(10):13455-65.
28. Harborg S, Cronin-Fenton D, Jensen MR, Ahern TP, Ewertz M, Borgquist S. Obesity and Risk of Recurrence in Patients With Breast Cancer Treated With Aromatase Inhibitors. JAMA Netw Open. 2023;6(10):e2337780.
29. Anwar SL, Cahyono R, Prabowo D, Avanti WS, Choridah L, Dwianingsih EK, et al. Metabolic comorbidities and the association with risks of recurrent metastatic disease in breast cancer survivors. BMC Cancer. 2021;21(1):590.
30. Zimbalist AS, Caan BJ, Chen WY, Mittendorf EA, Dillon DAR, Quesenberry C, et al. Metabolic abnormalities and survival among patients with non-metastatic breast cancer. BMC Cancer. 2022;22(1):1361.
31. Goss PE, Ingle JN, Pritchard KI, Ellis MJ, Sledge GW, Budd GT, et al. Exemestane versus anastrozole in postmenopausal women with early breast cancer: NCIC CTG MA.27--a randomized controlled phase III trial. J Clin Oncol. 2013;31(11):1398-404.
32. Cigler T, Richardson H, Yaffe MJ, Fabian CJ, Johnston D, Ingle JN, et al. A randomized, placebo-controlled trial (NCIC CTG MAP.2) examining the effects of exemestane on mammographic breast density, bone density, markers of bone metabolism and serum lipid levels in postmenopausal women. Breast Cancer Res Treat. 2011;126(2):453-61.

33. Bell LN, Nguyen AT, Li L, Desta Z, Henry NL, Hayes DF, et al. Comparison of changes in the lipid profile of postmenopausal women with early stage breast cancer treated with exemestane or letrozole. J Clin Pharmacol. 2012;52(12):1852-60.

34. Hozumi Y, Suemasu K, Takei H, Aihara T, Takehara M, Saito T, et al. The effect of exemestane, anastrozole, and tamoxifen on lipid profiles in Japanese postmenopausal early breast cancer patients: final results of National Surgical Adjuvant Study BC 04, the TEAM Japan sub-study. Ann Oncol. 2011;22(8):1777-82.

35. He T, Yang W, Zhang X, Li P, Yang D, Wu Y, et al. Comparative effectiveness of tamoxifen, toremifene, letrozole, anastrozole, and exemestane on lipid profiles in breast cancer patients: A network meta-analysis. Medicine (Baltimore). 2020;99(2):e18550.

36. Buss LA, Dachs GU. The Role of Exercise and Hyperlipidaemia in Breast Cancer Progression. Exerc Immunol Rev. 2018;24:10-25.

37. Cuzick J, Sestak I, Baum M, Buzdar A, Howell A, Dowsett M, et al. Effect of anastrozole and tamoxifen as adjuvant treatment for early-stage breast cancer: 10-year analysis of the ATAC trial. Lancet Oncol. 2010;11(12):1135-41.

Declarations

Ethics approval and consent to participate

This study was approved by the Ethics Committee of PUMCH (approval number: I-22PJ227). All patients signed informed consent.

Consent for publication

Not applicable.

Availability of data and materials

The data utilized and examined in this study are available upon reasonable request from the corresponding author.

Competing interests

The authors declare no competing interests.

Funding

This study was supported by National Natural Science Foundation of China (52173149), Beijing Natural Science Foundation (7222129) and National High Level

Hospital Clinical Research Funding (2022-PUMCH-B-038).

Authors' contributions

S.S. proposed study conception and design. Y.L., Z.D. and Y.W. performed material preparation, data collection and analysis. Y.L. and Z.D. performed statistical analysis. Y.L. and Z.D. wrote the first draft of the manuscript, and all authors commented on and revised previous versions of the manuscript. All the authors have read and approved the final manuscript.

Acknowledgments

The authors thank AiMi Academic Services (www.aimieditor.com) for English language editing and review services.
