# Supplementary material for: Lipid changes during endocrine therapy in early-stage breast cancer patients: A real-world study
Source: Lipids Health Dis. 2024 Jan 8;23:9. doi: 10.1186/s12944-024-02002-6 (PMC10773127; doi:10.1186/s12944-024-02002-6)
Supplement: Supplementary file 3 — Supplementary Material 3: Supplementary tables 1-5 [file 12944_2024_2002_MOESM3_ESM.docx]

Supplementary Table 1 Comparison of lipid profiles in AI and TOR breast cancer patients

|  | Time (month) | AI^&^ (SE) | TOR^&^ (SE) | *P*^#^ |
| --- | --- | --- | --- | --- |
| TC |  |  |  |  |
|  | 6 | 4.83 (0.06) | 4.69 (0.05) | 0.050 |
|  | 12 | 4.76 (0.05) | 4.65 (0.05) | 0.073 |
|  | 18 | 4.82 (0.05) | 4.64 (0.05) | 0.003 |
|  | 24 | 4.87 (0.05) | 4.69 (0.05) | 0.002 |
|  | 36 | 4.93 (0.05) | 4.71 (0.05) | ＜0.001 |
|  | 48 | 4.80 (0.06) | 4.81 (0.05) | 0.863 |
|  | 60 | 4.89 (0.08) | 4.96 (0.07) | 0.501 |
|  | 72 | 4.85 (0.12) | 4.91 (0.10) | 0.663 |
| TG |  |  |  |  |
|  | 6 | 1.32 (0.06) | 1.41 (0.06) | 0.312 |
|  | 12 | 1.23 (0.05) | 1.30 (0.04) | 0.272 |
|  | 18 | 1.21 (0.06) | 1.34 (0.05) | 0.077 |
|  | 24 | 1.18 (0.05) | 1.26 (0.04) | 0.124 |
|  | 36 | 1.18 (0.05) | 1.31 (0.04) | 0.031 |
|  | 48 | 1.12 (0.09) | 1.44 (0.08) | 0.007 |
|  | 60 | 1.16 (0.10) | 1.53 (0.09) | 0.004 |
|  | 72 | 1.15 (0.12) | 1.43 (0.10) | 0.070 |
| LDL-C |  |  |  |  |
|  | 6 | 2.90 (0.05) | 2.62 (0.05) | ＜0.001 |
|  | 12 | 2.84 (0.04) | 2.59 (0.04) | ＜0.001 |
|  | 18 | 2.89 (0.04) | 2.56 (0.04) | ＜0.001 |
|  | 24 | 2.92 (0.04) | 2.62 (0.04) | ＜0.001 |
|  | 36 | 2.96 (0.05) | 2.60 (0.04) | ＜0.001 |
|  | 48 | 2.87 (0.05) | 2.64 (0.05) | ＜0.001 |
|  | 60 | 2.94 (0.07) | 2.72 (0.06) | 0.011 |
|  | 72 | 2.95 (0.10) | 2.75 (0.09) | 0.117 |
| HDL-C |  |  |  |  |
|  | 6 | 1.33 (0.02) | 1.48 (0.02) | ＜0.001 |
|  | 12 | 1.36 (0.02) | 1.52 (0.02) | ＜0.001 |
|  | 18 | 1.38 (0.02) | 1.54 (0.02) | ＜0.001 |
|  | 24 | 1.42 (0.02) | 1.57 (0.02) | ＜0.001 |
|  | 36 | 1.44 (0.02) | 1.57 (0.02) | ＜0.001 |
|  | 48 | 1.44 (0.02) | 1.57 (0.02) | ＜0.001 |
|  | 60 | 1.47 (0.02) | 1.60 (0.02) | ＜0.001 |
|  | 72 | 1.44 (0.04) | 1.59 (0.03) | 0.001 |

^#^ Generalize Linear Mixed Models are used to compare the influence of different endocrine agents on blood lipids at each time point; the time of administration was taken as a classified variable.

^&^ The trend of serum lipid levels in different subgroups were described by model adjusted least-square means.

Supplementary Table 2 Baseline characteristics of premenopausal and postmenopausal breast cancer patients taking AI

| Variable | Postmenopausal AI (n=889) | Premenopausal AI (n=312) | t/X2 | *P*^#^ |
| --- | --- | --- | --- | --- |
| Age, mean（SD） | 60.89 (8.37) | 40.55 (6.17) | 39.32 | ＜0.001 |
| BMI（SD） | 24.74 (3.70) | 22.78 (3.11) | 6.68 | ＜0.001 |
| Surgery |  |  | 9.59 | 0.008 |
| Breast conserving | 362 (40.7) | 124 (39.7) |  |  |
| mastectomy | 509 (57.3) | 171 (54.8) |  |  |
| Adjuvant therapy |  |  |  |  |
| Target therapy | 132 (14.8) | 70 (22.4) | 9.504 | 0.002 |
| Chemotherapy | 299 (33.6) | 243 (77.9) | 182.6 | ＜0.001 |
| Comorbidities |  |  |  |  |
| hypertension | 144 (16.2) | 35 (11.2) | 4.52 | 0.034 |
| Coronary diseases | 12 (1.3) | 2 (0.6) | 0.49 | 0.316 |
| Diabetes | 36 (4.0) | 15 (4.8) | 0.327 | 0.568 |
| Baseline lipid profiles |  |  |  |  |
| TC, mean (SD) | 4.45 (0.52) | 4.32 (0.55) | 3.24 | 0.001 |
| TG, mean (SD) | 1.02 (0.33) | 0.82 (0.30) | 8.141 | ＜0.001 |
| LDL-C, mean (SD) | 2.48 (0.51) | 2.42 (0.51) | 1.661 | 0.097 |
| HDL-C, mean (SD) | 1.40 (0.26) | 1.44 (0.23) | 1.9 | 0.058 |

^#^ Comparisons of surgery type, adjuvant therapy and comorbidities are conducted using Pearson's chi-square or Fisher's exact; comparisons of age, BMI and baseline lipid profiles are conducted using Student's t-tests.

Supplementary Table 3 Comparison of lipid profiles in postmenopausal and premenopausal AI breast cancer patients at each medication time

|  | Time(month) | Postmenopausal AI^&^ (n=889) | Premenopausal  AI^&^ (n=312) | *P*^#^ |
| --- | --- | --- | --- | --- |
| TC |  |  |  |  |
|  | 6 | 4.97 (0.07) | 4.49 (0.12) | 0.001 |
|  | 12 | 4.86 (0.06) | 4.49 (0.10) | 0.002 |
|  | 18 | 4.93 (0.07) | 4.53 (0.11) | 0.001 |
|  | 24 | 4.92 (0.06) | 4.78 (0.10) | 0.243 |
|  | 36 | 5.00 (0.07) | 4.77 (0.12) | 0.078 |
|  | 48 | 4.84 (0.07) | 4.79 (0.14) | 0.741 |
|  | 60 | 4.91 (0.09) | 5.20 (0.21) | 0.218 |
|  | 72 | 4.87 (0.12) | 4.92 (0.26) | 0.865 |
| TG |  |  |  |  |
|  | 6 | 1.39 (0.06) | 1.37 (0.09) | 0.804 |
|  | 12 | 1.36 (0.05) | 1.15 (0.08) | 0.025 |
|  | 18 | 1.34 (0.05) | 1.13 (0.09) | 0.038 |
|  | 24 | 1.28 (0.05) | 1.13 (0.08) | 0.118 |
|  | 36 | 1.29 (0.05) | 1.15 (0.09) | 0.178 |
|  | 48 | 1.22 (0.05) | 1.06 (0.10) | 0.153 |
|  | 60 | 1.22 (0.06) | 1.20 (0.15) | 0.910 |
|  | 72 | 1.21 (0.11) | 1.11 (0.24) | 0.688 |
| LDL-C |  |  |  |  |
|  | 6 | 2.98 (0.06) | 2.51 (0.11) | ＜0.001 |
|  | 12 | 2.88 (0.06) | 2.51 (0.11) | ＜0.001 |
|  | 18 | 2.90 (0.06) | 2.58 (0.10) | 0.003 |
|  | 24 | 2.92 (0.05) | 2.77 (0.09) | 0.135 |
|  | 36 | 2.94 (0.06) | 2.79 (0.10) | 0.162 |
|  | 48 | 2.84 (0.06) | 2.76 (0.12) | 0.586 |
|  | 60 | 2.89 (0.08) | 3.15 (0.19) | 0.209 |
|  | 72 | 2.87 (0.11) | 2.87 (0.22) | 0.995 |
| HDL-C |  |  |  |  |
|  | 6 | 1.33 (0.02) | 1.30 (0.04) | 0.477 |
|  | 12 | 1.34 (0.02) | 1.37 (0.04) | 0.406 |
|  | 18 | 1.36 (0.02) | 1.38 (0.04) | 0.737 |
|  | 24 | 1.39 (0.02) | 1.45 (0.04) | 0.127 |
|  | 36 | 1.43 (0.02) | 1.43 (0.04) | 0.921 |
|  | 48 | 1.42 (0.02) | 1.47 (0.04) | 0.294 |
|  | 60 | 1.45 (0.03) | 1.50 (0.06) | 0.367 |
|  | 72 | 1.43 (0.04) | 1.42 (0.08) | 0.865 |

^#^ Generalize Linear Mixed Models are used to compare the influence of different endocrine agents on blood lipids at each time point; the time of administration was taken as a classified variable.

^&^ The trend of serum lipid levels in different subgroups were described by model adjusted least-square means.

Supplementary Table 4 Baseline characteristics of breast cancer patients taking different AIs

| Variable | ANA (n=723) | LET (n=298) | EXE (n=180) | F/X2 | *P*^#^ |
| --- | --- | --- | --- | --- | --- |
| Age, mean（SD） | 53.59 (11.77) | 59.14 (10.14) | 57.83 (13.26) | 27.89 | ＜0.001 |
| BMI（SD） | 24.02 (3.57) | 24.60 (3.51) | 24.16 (4.10) | 1.554 | 0.212 |
| Surgery |  |  |  | 16.77 | ＜0.001 |
| Breast conserving | 308 (42.6) | 92 (30.9) | 86 (47.8) |  |  |
| mastectomy | 396 (54.8) | 196 (65.8) | 88 (48.9) |  |  |
| Adjuvant therapy |  |  |  |  |  |
| Target therapy | 123 (17.0) | 42 (14.1) | 37 (20.6) | 3.40 | 0.183 |
| Chemotherapy | 329 (45.5) | 114 (38.3) | 99 (55.0) | 12.81 | 0.002 |
| Comorbidities |  |  |  |  |  |
| hypertension | 114 (15.8) | 39 (13.1) | 26 (14.4) | 1.231 | 0.54 |
| Diabetes | 38 (5.3) | 7 (2.3) | 6 (3.3) | 4.82 | 0.09 |
| Baseline lipid profiles |  |  |  |  |  |
| TC, mean (SD) | 4.42 (0.52) | 4.40 (0.55) | 4.38 (0.56) | 0.221 | 0.802 |
| TG, mean (SD) | 0.93 (0.33) | 1.03 (0.33) | 0.95 (0.35) | 5.606 | 0.004 |
| LDL-C, mean (SD) | 2.47 (0.49) | 2.44 (0.55) | 2.45 (0.54) | 0.192 | 0.825 |
| HDL-C, mean (SD) | 1.42 (0.26) | 1.39 (0.26) | 1.42 (0.23) | 1.316 | 0.269 |

^#^ Comparisons of surgery type, adjuvant therapy and comorbidities are conducted using Pearson's chi-square or Fisher's exact; comparisons of age, BMI and baseline lipid profiles are conducted using Student's t-tests.

Supplementary Table 5 Comparison of lipid profiles in patients taking different AIs

|  | TC (β^#^, 95%CI) | TG (β^#^, 95%CI) | LDL-C  (β^#^, 95%CI) | HDL-C  (β^#^, 95%CI) |
| --- | --- | --- | --- | --- |
| AI |  |  |  |  |
| ANA | 0 | 0 | 0 | 0 |
| LET | 0.133  (-0.029,0.295) | 0.091  (-0.011, 0.193) | 0.079  (-0.065,0.222) | -0.038  (-0.095,0.019) |
| EXE | -0.013  (-0.195,0.168) | -0.048  (-0.160, 0.064) | -0.032  (-0.193,0.129) | 0.053  (-0.013,0.119) |
| Time | 0.089（0.074,0.104） | 0.052 (0.042,0.063) | 0.066 (0.053,0.079) | 0.016 (0.011,0.020) |
| Time*AI |  |  |  |  |
| ANA*time | 0 | 0 | 0 | 0 |
| LET*time | -0.046  (-0.074, -0.018) | -0.026  (-0.045, -0.007) | -0.019  (-0.043,0.005) | -0.006  (-0.014,0.002) |
| EXE*time | -0.073  (-0.109, -0.038) | -0.044  (-0.068, -0.020) | -0.018  (-0.048,0.013) | -0.034  (-0.045, -0.024) |
| Age | 0.003  (-0.002,0.008) | 0.007  (0.004,0.010) | -0.001  (-0.005,0.004) | -0.001  (-0.002,0.002) |
| BMI | -0.019  (-0.033, -0.004) | 0.030  (0.020,0.040) | -0.001  (-0.013,0.013) | -0.023  (-0.028, -0.017) |
| Surgery |  |  |  |  |
| Breast conserving | 0 | 0 | 0 | 0 |
| Mastectomy | 0.194 (0.088,0.300) | 0.096  (0.024,0.168) | 0.175  (0.080,0.270) | -0.034  (-0.074,0.005) |
| Adjuvant therapy |  |  |  |  |
| Target therapy | 0.072 (-0.086,0.230) | 0.028  (-0.077,0.134) | 0.025  (-0.116,0.166) | 0.010  (-0.049,0.069) |
| Chemotherapy | 0.035 (-0.092,0.163) | -0.041  (-0.127,0.046) | 0.072  (-0.042,0.186) | -0.002  (-0.049,0.045) |
| Comorbidities |  |  |  |  |
| Hypertension | -0.037  (-0.185,0.110) | -0.023  (-0.123,0.076) | -0.008  (-0.140,0.124) | -0.007  (-0.062,0.047) |

^#^ Generalize Linear Mixed Models are used to evaluate the overall trend of blood lipid changes; the time of administration was taken as a continuous variable.
